# Microwave-Assisted Depolymerization of Polyurethane and Polyisocyanurate Foams with Diethylene Glycol and Epoxidized Tall Oil-Based Polyol

**DOI:** 10.3390/polym18202498

**Published:** 2026-10-13

**Authors:** Beatrise Sture-Skela, Darta Lina Eihe, Ralfs Pomilovskis, Hynek Beneš, Ugis Cabulis

**Affiliations:** 1 Polymer Laboratory, Latvian State Institute of Wood Chemistry, Dzerbenes Street 27, LV-1006 Riga, Latvia; beatrise.stureskela@kki.lv (B.S.-S.); darta.eihe@kki.lv (D.L.E.); ralfs.pomilovskis@kki.lv (R.P.); 2 Institute of Macromolecular Chemistry, Czech Academy of Sciences, Heyrovskeho nam. 2, 162 00 Prague 6, Czech Republic; benesh@imc.cas.cz

**Keywords:** polyurethane, polyisocyanurate, recycling, depolymerization, tall oil, microwave

## Abstract

Polyurethane (PU) foams have been widely used for decades for various applications, and this high demand for PU products, naturally, increases the amount of waste; therefore, effective recycling methods must be explored. Depolymerization via glycolysis has already been performed on an industrial scale with different glycols. Meanwhile, our research focuses on the incorporation of epoxidized tall oil-based (ETOFA) polyol into the depolymerization process. Even though glycolysis does not require harsh conditions, novel depolymerization methods should still be investigated, focusing on shortening reaction time, for example, microwave (MW)-assisted reaction. In our research, four PU and polyisocyanurate (PIR) foams from bio-based and petrochemical polyols with different isocyanate indexes (110 and 400) were depolymerized with diethylene glycol (DEG) and epoxidized tall oil fatty acids/diethanolamine (ETOFA/DEOA) polyol. MW-assisted depolymerization was achieved within 5–10 min under the tested conditions, while conventional depolymerization required up to 5.5 h. Recycled polyols obtained with DEG showed OH values of 542–619 mg KOH/g, while partial substitution of DEG with ETOFA/DEOA resulted in lower OH values of 320–450 mg KOH/g, reflecting the lower OH value of the ETOFA/DEOA polyol. Incorporation of ETOFA/DEOA increased the viscosity of the recycled polyols, which should be considered for further processing.

## 1. Introduction

Polyurethane (PU) is the sixth most used synthetic polymer worldwide, with the market size reaching USD 88 billion in 2025 and an expected compound annual growth rate of 5.6% through 2034 [1,2]. Rigid PU foams are widely used as thermal insulation materials in construction, automotive, furniture, and cold-chain applications due to their low density, excellent thermal conductivity, and favorable mechanical properties [3]. Polyisocyanurate (PIR) foams, characterized by a higher isocyanate index (NCO index above 150), offer enhanced fire resistance compared to conventional PU foams and are commonly used in building insulation panels [4]. The growing volume of PU and PIR foam waste, including post-consumer products and manufacturing scraps, poses significant environmental challenges. Landfilling is problematic due to volume and low density, while incineration raises concerns about toxic gas emissions, particularly from isocyanate-derived compounds [2].

Since demand and applications of PUs and PIRs do not decrease, naturally, waste products are becoming a bigger issue; therefore, effective recycling methods must be investigated. Among other recycling approaches, like aminolysis, acidolysis, methanolysis, hydrolysis, etc., chemical recycling through glycolysis has emerged as the most promising method for industrial upscaling [2]. Glycolysis involves transesterification reactions between the ester part of urethane bonds in PU waste and the hydroxyl groups of a glycolytic agent, producing mixed-structure polyols that can be reused in new foam formulations [5]. The use of other recycling approaches might be limited in the future, since most components used for these processes are petrochemical, which have a negative impact on the environment.

Diethylene glycol (DEG) is the most commonly used glycolysis agent for rigid PU foam depolymerization. It acts as a solvolysis agent that cleaves urethane bonds at elevated temperatures (150–220 °C), producing a liquid polyol product with suitable hydroxyl values for re-incorporation into new foam systems [2,5,6]. The reaction is typically conducted with metal-based catalysts such as acetates or alkali metal hydroxides, which accelerate the transesterification process, but some conventional degradation catalysts might have poor dispersion [2,5]. Successful results have been obtained by using a core–shell nanoscale titanium catalyst, which has better dispersion in depolymerizing agents [7]. Conventional glycolysis of rigid PU foams typically requires 4–6 h of heating at 180–220 °C. Microwave (MW) irradiation offers a transformative alternative by providing volumetric heating that is far more efficient than surface conduction [2]. MW-assisted glycolysis dramatically shortens reaction time compared to conventional heating methods, offering a substantial improvement in processing efficiency. This approach also delivers notable energy savings (in some cases, up to 45–50%), consuming significantly less energy than conventional heating processes [2,8]. In terms of product quality, materials produced through MW glycolysis tend to show viscosity, hydroxyl value, and mechanical performance that are comparable to or better than conventional methods [5,9]. MW-assisted depolymerization has already been proven successful for recycling of other polymer materials like polyethylene terephthalate (PET) [10,11,12].

Tall oil, a by-product of the Kraft pulping process, represents a promising renewable feedstock for polyol synthesis in rigid PU and PIR foam production. Tall oil fatty acids (TOFAs) can be converted into polyols through epoxidation (ETOFAs), followed by oxirane ring opening with polyfunctional alcohols, yielding bio-polyols with controlled functionality and hydroxyl values [13]. Recent research has demonstrated that rigid PU foams produced with tall oil-based polyols can achieve competitive properties, including favorable apparent densities, high closed-cell content, strong compressive strength, and low thermal conductivity [14,15]. These bio-based foams can incorporate a substantial proportion of renewable material content while meeting the performance requirements for thermal insulation applications [16].

So far, depolymerization of PU foams has been performed mostly with petrochemical glycols [17,18]. However, several studies using renewable reagents, such as coconut oil waste fraction, are also emerging [19,20]. Our approach presented in this study—MW-assisted glycolysis of PU/PIR waste depolymerization with ETOFA-based polyols—represents a circular economy concept that addresses both waste management and renewable feedstock integration challenges in the polyurethane industry. Since DEG is a small-chain alcohol, it has high dielectric loss ε″ (>14.0), and it efficiently converts MW energy into thermal energy and can more easily dissolve foam [21]. As ETOFA polyols mainly consist of long-chain fatty acids, the resulting polyol used for depolymerization also has long chains and consequently lower ε″.

In this study, MW-assisted glycolysis of PU and PIR foams is performed with DEG and a mixture of DEG and ETOFA polyol. Although glycolysis has already been extensively studied, so far, no research involving tall oil-based polyols has been revealed. Obtained recycled polyols contain urethane groups and will be used as precursors for the development of Michael polymers.

## 2. Materials and Methods

### 2.1. Materials

DEG (purity ≥ 99%) for depolymerization was purchased from Chempur, Germany. Meanwhile, ETOFA polyol was obtained through amidation with diethanolamine (DEOA) in our previous research [18], where synthesis of polyols was carried out in relatively mild reaction conditions, without toxic organic solvents or any harmful by-products, in accordance with “Green Chemistry” principles.

Catalyst potassium hydroxide KOH (ACS reagent, pellets, ≥85%) was purchased from Sigma Aldrich, Schnelldorf, Germany.

Reagents for polyol analysis (acid value, hydroxyl value, moisture content): 4-(dimethylamino)pyridine, reagent plus, ≥99%; acetic anhydride, puriss, ≥99%; methanol, puriss p.a., ACS reagent, ≥99.8%; N,N-dimethylformamide, ACS reagent, ≥99.8%, water content ≤150 ppm; potassium hydroxide, ACS reagent, pellets, ≥85%; N,N-dimethylformamide, biotech. Grade, ≥99.9%, purchased from Sigma Aldrich, Schnelldorf, Germany; Hydranal Coulomat CG catholyte and anolyte solutions both purchased from Sigma Aldrich, Seelze, Germany.

Tetrahydrofuran (for GPC/SEC), puriss p.a., 99.9%, was purchased from Lachner, Neratovice, Czech Republic.

### 2.2. Methods

#### 2.2.1. PU and PIR Foams for Depolymerization

Foams from bio-based and petrochemical polyols for depolymerization were prepared using formulations developed in our previous studies [22,23]. Four different foams were used for depolymerization, denoted 110bio, 110petro, 400bio and 400petro, where number in sample ID indicates isocyanate index (110 or 400); respectively, 110bio being foam from bio-based polyols with isocyanate index of 110, 110petro—foam from petrochemical polyols with isocyanate index of 110, 400bio—foam from bio-based polyols with isocyanate index of 400, and 400petro—foam from petrochemical polyols with isocyanate index of 400. Afterwards, foams were cut into 1.5–2 cm thick plates, and each plate was pressed with hot press (180 °C) at 50 kN for 1 min, further at 100 kN for 1 min, at 200 kN for 1 min and at 40 kN for 1 min. Pressed foams were cut into smaller pieces (around 5 × 5 mm). More extensive description of foams has been presented in our previous research [24].

#### 2.2.2. Microwave-Assisted Depolymerization

The first series of MW-assisted depolymerization took place in RV EKO L1 microwave oven adjusted for laboratory usage, which has max power of 1200 W. For depolymerization process, a 3-neck round-bottom flask (250 mL) was used, which was equipped with a mechanical stirrer and a dephlegmator. The foam sample was weighed into a flask with DEG polyol; catalyst KOH was used for depolymerization of foams with isocyanate index of 400—400bio and 400petro. Depolymerization process took place at two different power levels of microwave oven, 80% and 60%. Firstly, depolymerization was performed for 10 min; afterwards, trials to obtain recycled polyols in 5 min (at both power levels 80% and 60%) were started. Unfortunately, not all polyols were obtained in such conditions; therefore, in some cases, duration of depolymerization was increased up to 7.5 min (all conditions are presented further in Results and Discussion). At first, depolymerization was performed with only DEG at mass ratio of 1:1.5 (foam/DEG), and catalyst was added as 1 weight % from foam mass. Recycled polyols were analyzed by determining acid value, hydroxyl value, viscosity and moisture content. Their chemical structure was analyzed with FTIR and GPC/SEC.

Further depolymerization was performed with DEG and ETOFA/DEOA (further abbreviated as ED) polyol at mass ratio 1:0.75:0.75 (foam/DEG/ED); catalyst was also added to depolymerization of foams with isocyanate index of 400 (400bio and 400petro). Depolymerization conditions (duration and power) were kept the same. Recycled polyols were characterized as previously mentioned.

The second series of MW decompositions were performed in monomodal MW reactor Discover SP Microwave synthesizer (CEM Corporation) operating at 2450 MHz frequency. The components of reactive mixtures were placed into a 50 mL glass vessel together with a magnetic stirrer and then sealed tightly with a PTFE-coated silicone septum. The reaction mixture was heated using fixed power mode (150 W) and the temperature was monitored using infrared thermometer. These experiments allowed us to obtain data, which are more comparable to larger pilot-scale MW-assisted depolymerization.

#### 2.2.3. Characterization of Recycled Polyols

All titrations followed relevant testing standards. Acid value and hydroxyl value were measured per ISO 2114:2000 and ISO 4629-2:2016, respectively, and three parallel measurements of each sample were taken. Water content was determined by Karl Fischer titration on a Denver Instrument Model 275 KF automatic titrator (Denver Instrument, Bohemia, NY, USA).

Rheological behavior was assessed with an Anton Paar Modular Compact Rheometer MCR 92 (Anton Paar, Graz, Austria) fitted with a cone-plate geometry and a 48 µm gap, applying shear rate ramps from 1 to 1000 s^−1^. Tests were run at a constant temperature of 25 °C. Three parallel measurements of each sample were taken, and error margins were determined using Student’s t-distribution, applying a coefficient corresponding to a 95% confidence interval.

FTIR spectra were recorded on a Thermo Scientific Nicolet iS50 spectrometer (Thermo Fisher Scientific, Waltham, MA, USA) at 4 cm^−1^ resolution over 32 scans, using attenuated total reflectance with a diamond crystal.

GPC/SEC analysis was performed using an Agilent Infinity 1260 HPLC system (Agilent Technologies, Inc., Santa Clara, CA, USA) equipped with a refractive index detector and a multi-angle static light scattering (miniDAWN) detector. Two PLgel Mixed-E columns (particle size: 3 μm, 300 × 7.5 mm) connected in series were used for separation. Tetrahydrofuran (THF, ≥99.9%, HPLC grade, inhibitor-free, Supelco) was used as the mobile phase at a flow rate of 1 mL/min. The refractive index detector was maintained at 35 °C. Samples were prepared in THF at a concentration of 10 mg/mL, and an injection volume of 100 μL was used.

## 3. Results

At first, depolymerization was performed using only DEG; after that, depolymerization was achieved, partially substituting DEG with ETOFA/DEOA (ED) polyol. Recycled polyols were obtained through MW-assisted depolymerization, and the characteristics of these polyols were compared to recycled polyols obtained through conventional heating [24]. This helps to evaluate the MW process and emphasize advantages.

In Table 1, the conditions (catalyst, foam/DEG mass ratio, power level) of depolymerization with DEG are presented. It must be pointed out that depolymerization of foams with isocyanate index 110 (110bio and 110petro) occurred without the presence of catalyst KOH. The microwave experiments were exploratory and not designed as a factorial optimization study. Microwave power, reaction time, and final temperature were not independently controlled, and temperatures were measured only at the end of the process. Therefore, observed differences are discussed as differences between tested conditions rather than isolated effects of individual parameters.

In Table 2, conditions (catalyst, foam/DEG/ED mass ratio, power level) of depolymerization with DEG and ED polyol are presented. Also, in this case, depolymerization of foams with isocyanate index 110 (110bio and 110petro) occurred without the presence of catalyst KOH.

### 3.1. OH Values

One of the key characteristics of recycled polyols is OH value, as polyols are commonly used in the production of PU and PIR foams, and the amount of isocyanate required in foam formulations depends directly on this parameter. Our further research focuses on the development of Michael donor and acceptor components, for which the OH value is a critical parameter influencing successful synthesis of these components [25,26]. Acid value and moisture content of recycled polyols are presented in Supplementary Materials (Table S1).

**Figure 1:**
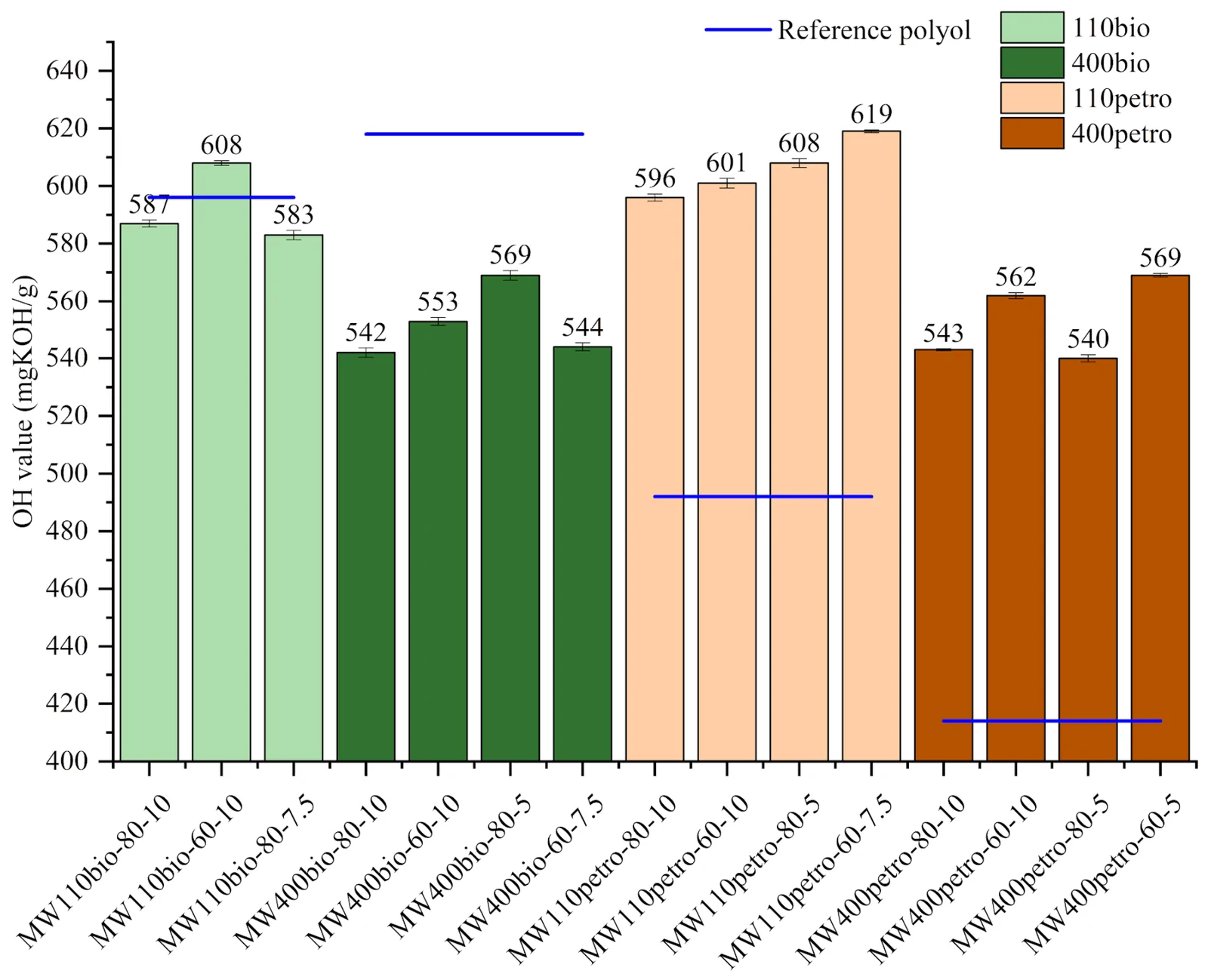
OH values of recycled polyols depolymerized with DEG (reference polyol is polyol obtained in conventional depolymerization with DEG [24]).

OH values of recycled polyols depolymerized with DEG are presented in Figure 1.

Even though the variation between OH values is not significant, recycled polyols obtained in the presence of the KOH catalyst from foams with an isocyanate index of 400 (400bio and 400petro) exhibited slightly lower OH values than the corresponding reference polyols. In our previous research, depolymerization of foams 400bio and 400petro in the presence of an acidic catalyst (zinc acetate) did not occur under conventional heating conditions [24]. Similar observations have been reported by Dulyanska et al. While acidic and basic catalysts generally produce recycled polyols with comparable OH values, acidic catalysts demonstrate higher yields of polyols compared to basic catalysts [27]. Gu et al. studied the impact of KOH and ethylene glycol–titanium on depolymerization of foams, where KOH was found to be less efficient in breaking carbamate bonds of PU foams—this results in more end- and branched hydroxyl groups and consequently lower OH values, which correlates with our results [7]. In contrast, Nikje et al. demonstrated that acetate catalysts exhibit lower catalytic activity during MW-assisted glycolysis, leading to longer foam dissolution times, whereas KOH enabled more efficient depolymerization [5].

Recycled polyols from foams 110bio and 110petro were successfully obtained without a catalyst, most likely because their lower crosslinking density facilitated the depolymerization process. Although these recycled polyols exhibit slightly higher OH values than the corresponding reference polyols, they are still optimal for further processing. Overall, several recycled polyols had higher OH values compared to reference polyols (see Figure 1); however, the MW-assisted depolymerization required a substantially shorter reaction time. Further systematic experiments would be required to determine the individual effects of the power level and reaction duration on these values. Nonetheless, values can be potentially altered by optimizing depolymerization conditions such as power level and reaction duration. Kiss et al. compared glycolysis of foams in atmospheric pressure, autoclave and MW-assisted depolymerization, and all three methods showed similar results (close OH values), but the duration of MW-assisted depolymerization was considerably reduced [28].

The next stage of this study involved partially substituting DEG with ED polyol.

Since ED polyol itself has an OH value of 474 mgKOH/g (in comparison, DEG has ~1000 mgKOH/g), naturally, recycled polyols depolymerized with DEG and ED exhibited lower OH values than those produced with DEG alone. In contrast to previous results, here, no significant impact of the catalyst KOH can be depicted, although it was used only for depolymerization of foams 400bio and 400petro.

**Figure 2:**
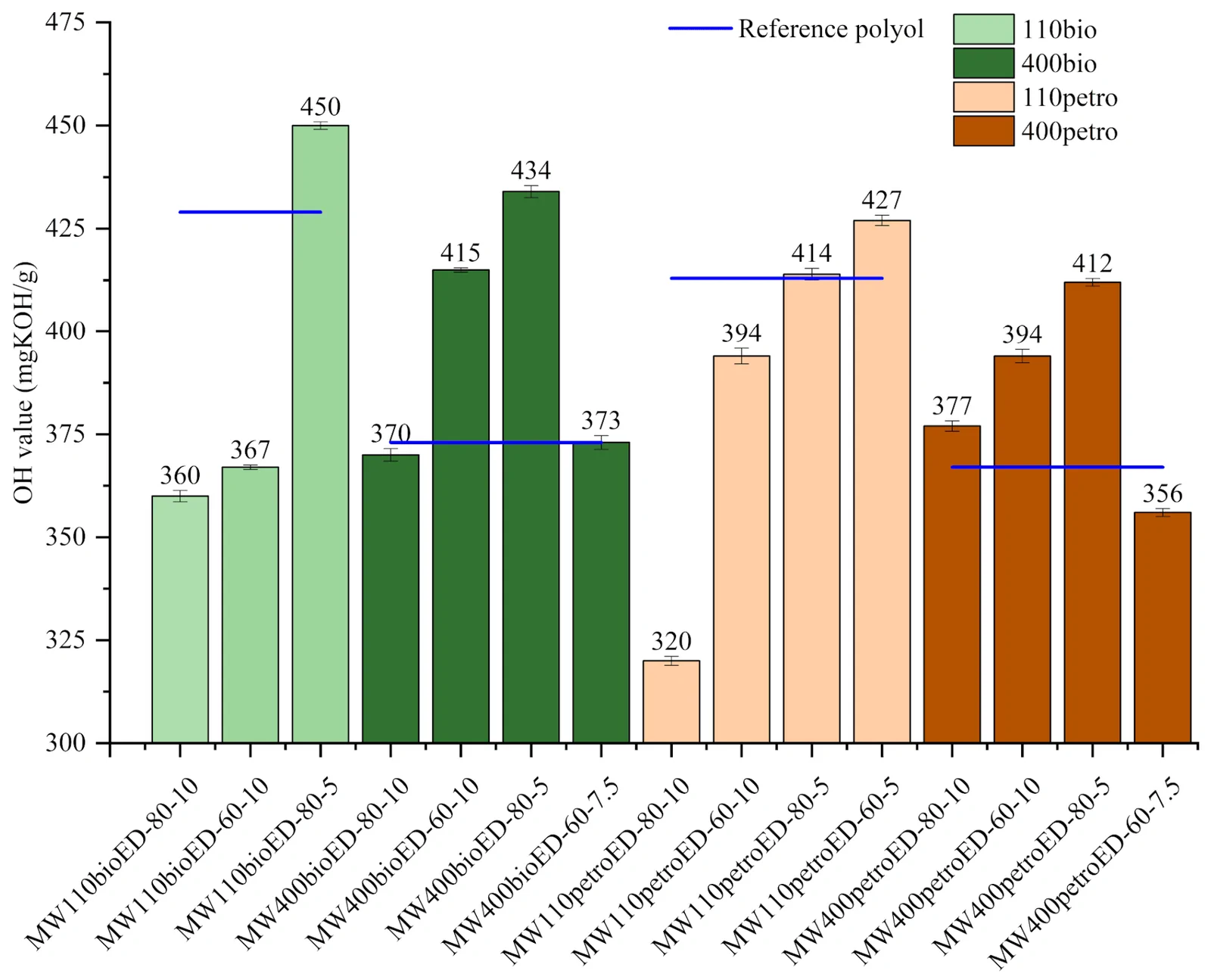
OH values of recycled polyols depolymerized with DEG+ED (reference polyol is polyol obtained in conventional depolymerization with DEG+ED [24]).

Based on the data presented in Figure 2, OH values are less distinct from the values of reference polyol obtained in conventional depolymerization. This demonstrates that MW-assisted depolymerization can achieve comparable product quality while substantially reducing reaction time. As previously mentioned, further optimization of depolymerization conditions is the key to more favorable values.

Overall, the evaluation of all recycled polyols produced using both DEG and DEG+ED confirms that MW-assisted depolymerization yields recycled polyols with OH values comparable to those obtained via conventional depolymerization. Although most recycled polyols exhibited slightly higher OH values than the reference materials, the differences were relatively small. Under the tested conditions, MW-assisted depolymerization produced recycled polyols with comparable OH values within substantially shorter reaction times than conventional depolymerization.

### 3.2. Viscosity

Viscosity is also one of the key characteristics of polyols because it directly influences their processability, including pumping, mixing with isocyanates, and the homogeneity of PU and PIR formulations. Therefore, it is an important parameter for evaluating the suitability of recycled polyols for further applications. For most recycled polyols, viscosity varied by approximately 2–5% over the investigated shear-rate range of 1–1000 s^−1^, indicating approximately Newtonian behavior, which is very favorable and is an advantageous characteristic to the industry.

**Figure 3:**
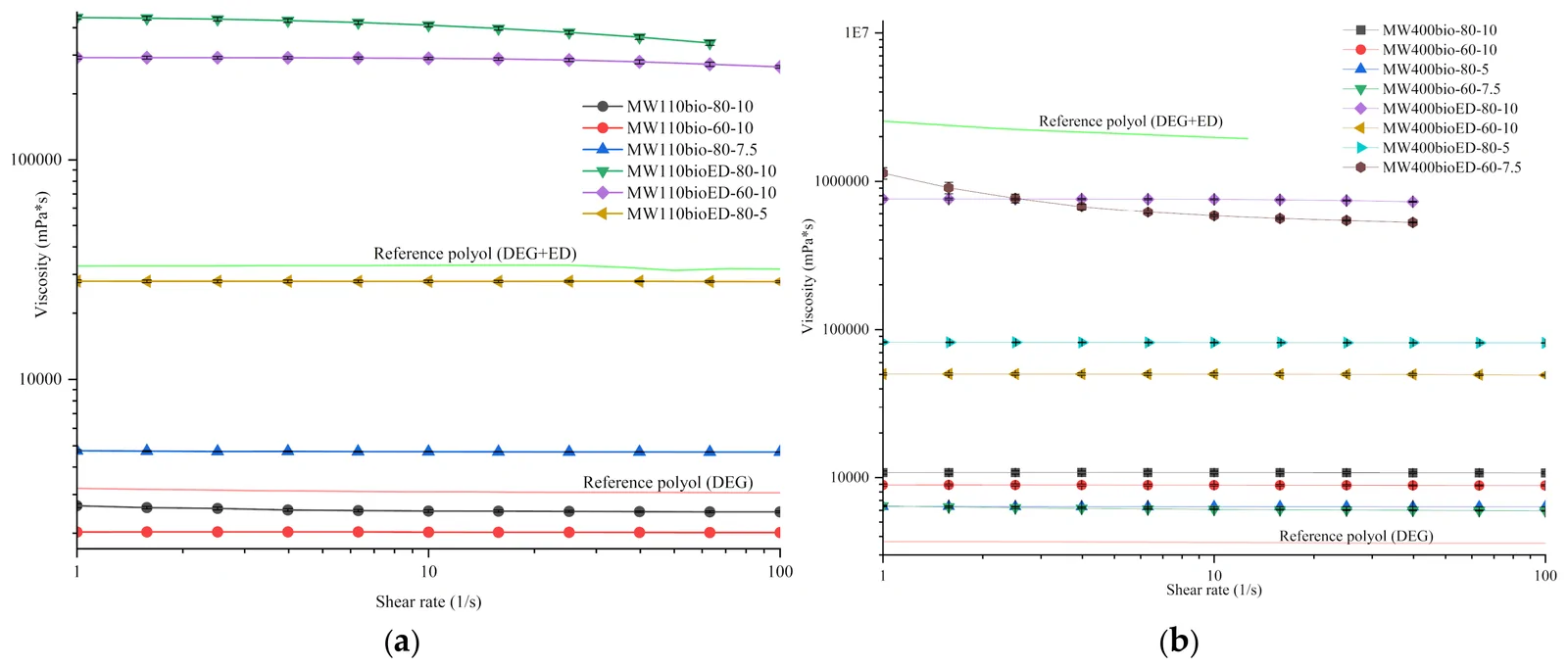
Rheological measurements of recycled polyols (**a**) 110bio and (**b**) 400bio (reference polyol is polyol obtained in conventional depolymerization, respectively, with DEG or DEG+ED [24]).

Graphs in Figure 3 represent rheological measurements of recycled polyols from foams 110bio (a) and 400bio (b). It can be observed that, in both cases, recycled polyols depolymerized with DEG+ED have higher viscosities, which is evident because ED polyol itself has high viscosity of ~104 000 mPa·s, but DEG has very low viscosity of ~40 mPa·s. A similar trend was also observed with recycled polyols obtained via conventional depolymerization [24]. Lower viscosity can also be attributed to effective coupling between the catalyst and DEG, which stimulates high dielectric heating and transesterification, and the desired viscosity can be achieved faster than in conventional heating [2].

In the case of recycled polyols from foam 110bio, the impact of duration of the reaction can also be depicted. Samples MW110bio-80-10 and MW110bio-60-10 (obtained in 10 min reaction) exhibit lower viscosities than samples prepared in a shorter reaction (see Figure 3a). This suggests that extending the reaction time promotes a greater extent of depolymerization and dissolution of foam particles. However, the opposite effect can be observed when depolymerization is performed with DEG+ED polyol, indicating that the presence of the highly viscous ED polyol becomes the dominant factor governing the rheological behavior. Catalyst type also has an impact on the viscosity. In research performed by Gu et al., a catalyst with higher surface area had a higher catalytic effect, whereas traditional catalysts like KOH may have lower catalytic efficiency, although this could be improved by increasing the concentration of the catalyst [7]. In the present work, KOH provided efficient depolymerization of the highly crosslinked foams with an isocyanate index of 400, confirming its suitability for MW-assisted glycolysis.

**Figure 4:**
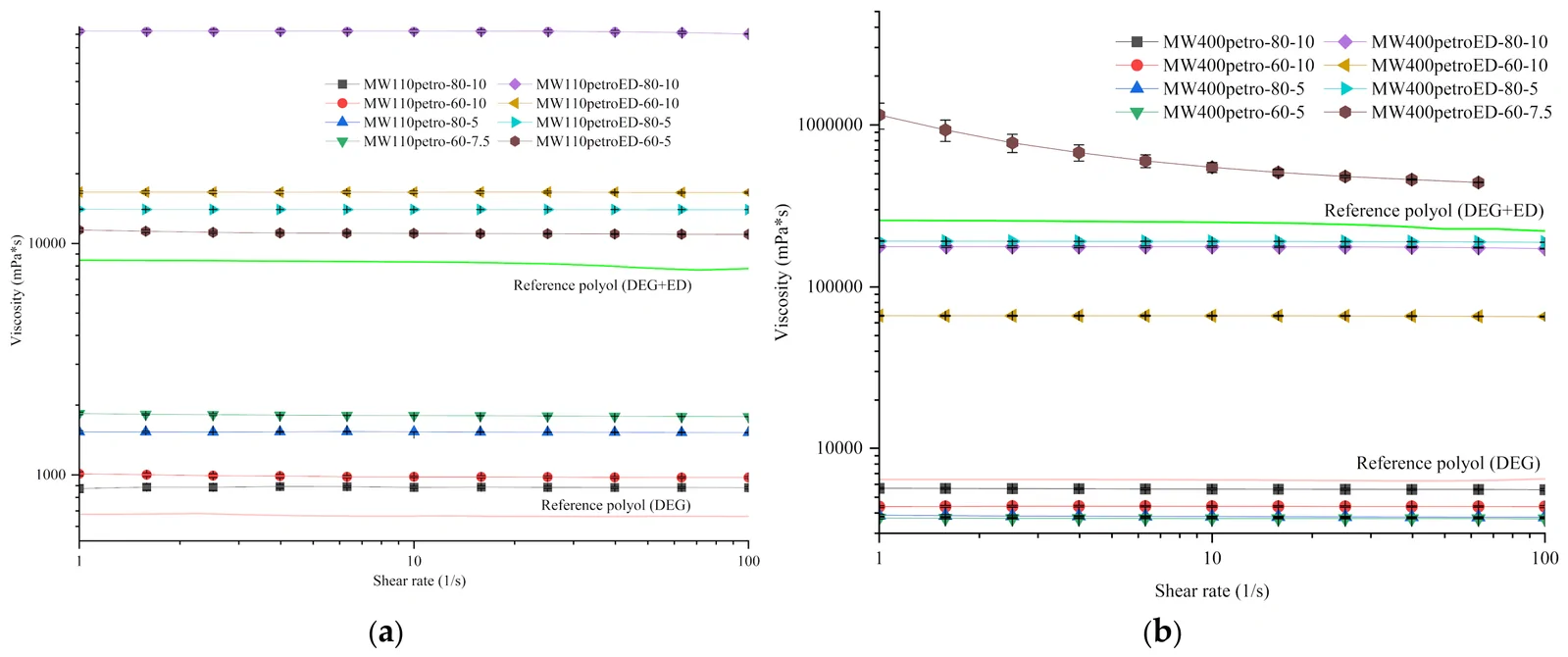
Rheological measurements of recycled polyols (**a**) 110petro and (**b**) 400petro (reference polyol is polyol obtained in conventional depolymerization, respectively, with DEG or DEG+ED [24]).

Similar trends can also be depicted in viscosity data of recycled polyols from foams 110petro and 400petro (see Figure 4)—in all cases, recycled polyols prepared using only DEG exhibited lower viscosities than those obtained using DEG+ED mixture, confirming that the viscosity of the glycolysis medium has a pronounced influence on the rheological properties of the recovered polyols.

A comparison of all recycled polyols revealed that the viscosity depended on both the isocyanate index of the original foam and the composition of the glycolysis medium; however, this cannot be assigned only to reaction time because power, time and temperature were not independently controlled. For recycled polyols obtained using only DEG from foams with an isocyanate index of 110 (110bio and 110petro), viscosity decreased with increasing reaction time. The opposite effect is visible in the case of recycled polyols depolymerized with DEG from foams with an isocyanate index of 400 (400bio and 400petro) and recycled polyols depolymerized with DEG+ED polyol from foams with an isocyanate index of 110 (110bio and 110petro)—viscosity increases when the duration of the reaction is higher. Meanwhile, recycled polyols depolymerized with DEG+ED polyol from foams with an isocyanate index of 400 (400bio and 400petro) show no association between viscosity and duration of reaction. These findings indicate that the rheological properties of the recycled polyols are governed by the combined effects of foam structure, glycolysis medium, catalyst, and reaction time, rather than by a single processing parameter.

Overall, the rheological measurements demonstrate that MW-assisted depolymerization produces recycled polyols with favorable flow behavior and viscosities comparable to those previously obtained by conventional heating. Although the incorporation of ED polyol increases viscosity because of its inherently high viscosity, most recycled polyols retained Newtonian behavior, indicating their suitability for further processing.

### 3.3. FTIR Analysis

The chemical structure of recycled polyols was analyzed with FTIR and GPC/SEC (see Section 3.4).

FTIR analysis was performed by comparing initial foam, pure DEG, ED polyol and recycled polyol. The active process of depolymerization can be analyzed via changes in peaks, which are characteristic to urethane groups—carbonyl C=O stretching at ~1720 cm^−1^, -N-H bending at ~1515 cm^−1^ and C-N stretching at ~1600 cm^−1^ [7,27,29].

**Figure 5:**
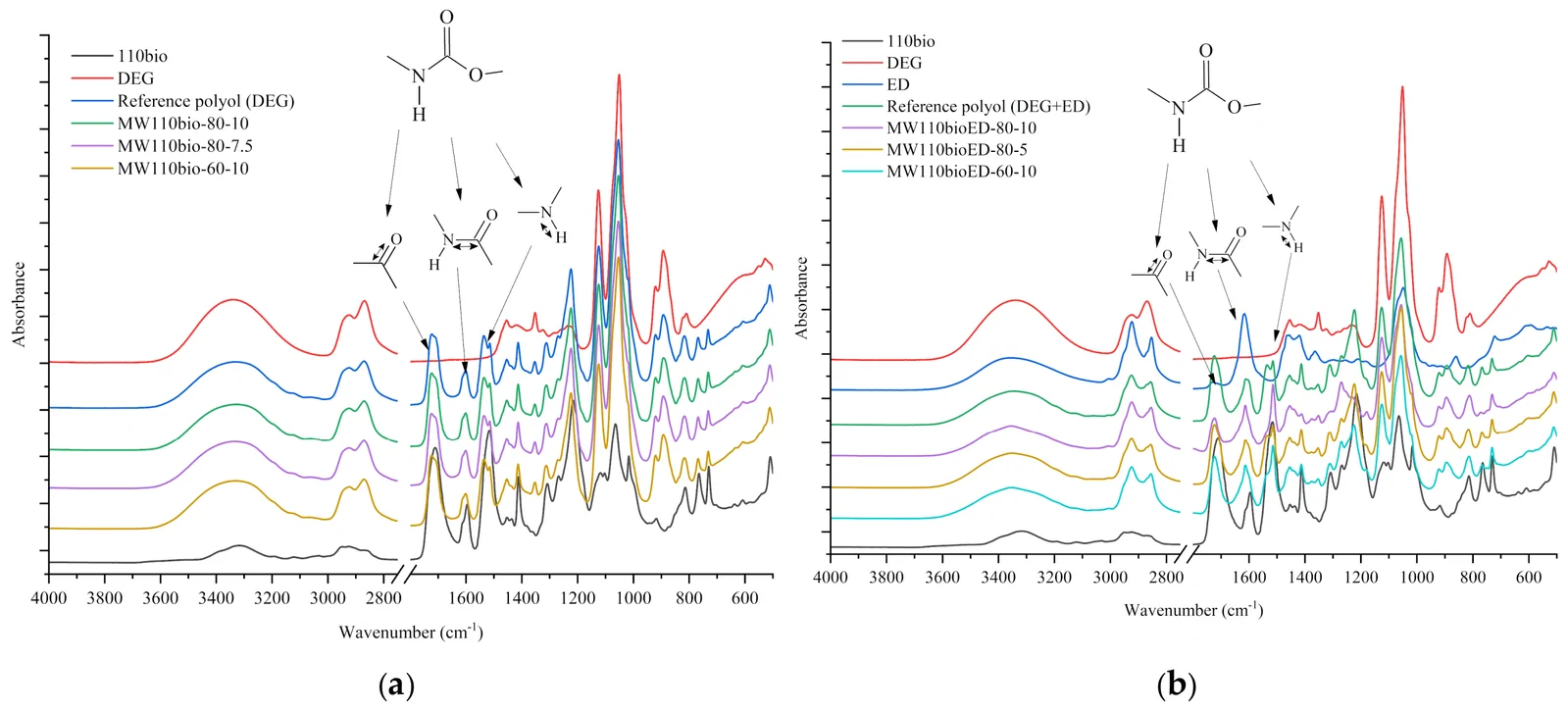
FTIR spectra of recycled polyols from foam 110bio (**a**) depolymerized with DEG and (**b**) depolymerized with DEG+ED (reference polyol is polyol obtained in conventional depolymerization, respectively, with DEG or DEG+ED [24]).

Representative FTIR spectra of recycled polyols obtained from foam 110bio are presented in Figure 5. Compared with the initial foam, the recycled polyols exhibited a noticeable reduction in the intensity of the characteristic urethane absorption bonds, consistent with the cleavage of the urethane linkages during glycolysis and, consequently, successful depolymerization. However, no significant changes can be depicted comparing recycled polyols obtained during different depolymerization conditions, therefore indicating that the investigated depolymerization conditions have a limited impact on peak size, and depolymerization can effectively occur during more mild conditions.

Relative sizes of these peaks were also measured, and these data, in addition to spectra of other recycled polyols, are presented in Supplementary Materials (Table S3 and Figures S1–S4).

A decrease in the absorbance maximum at ~3300 cm^−1^ compared to pure DEG and ED polyol can be observed in the spectra of each recycled polyol. This band corresponds primarily to O–H stretching vibrations and is consistent with the lower hydroxyl values determined by titration. Thus, the FTIR analysis supports the results obtained from the chemical characterization of the recycled polyols.

**Figure 6:**
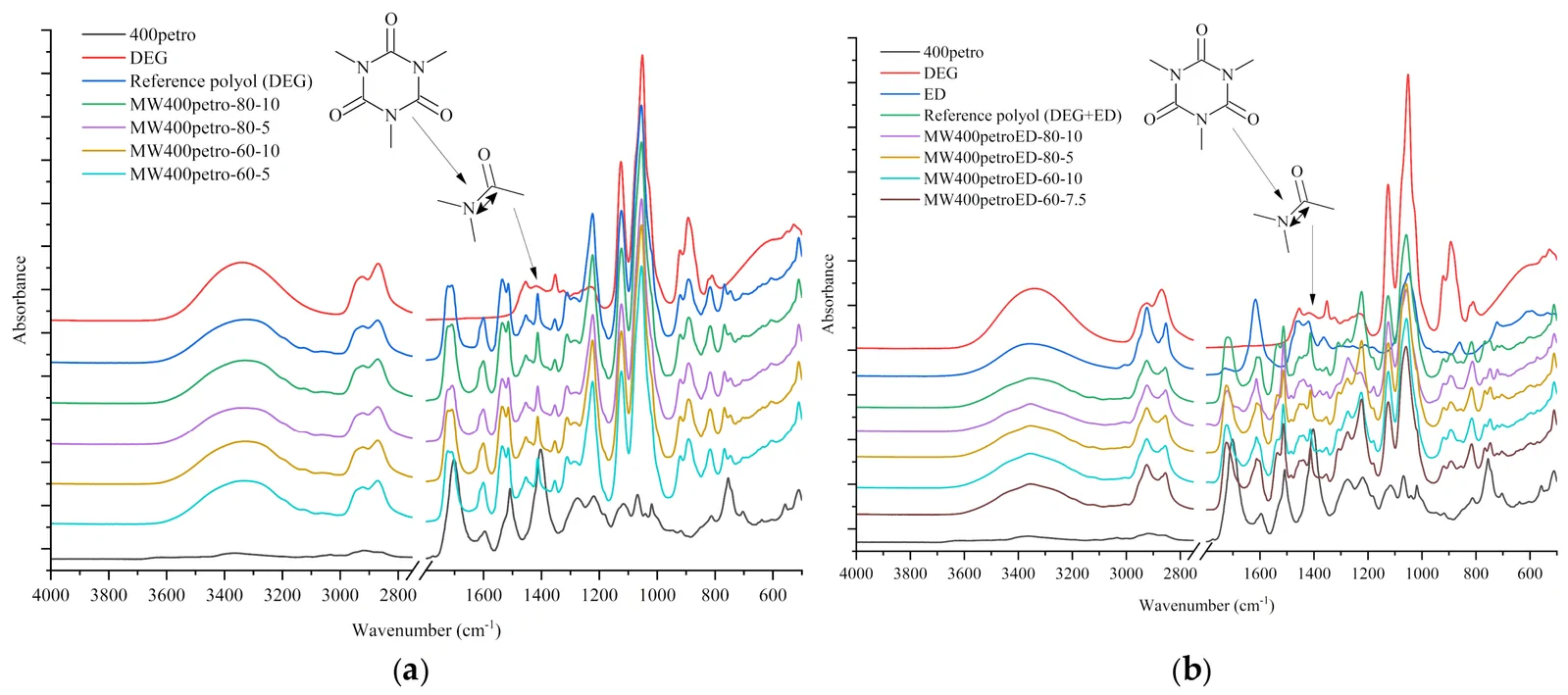
FTIR spectra of recycled polyols from foam 400petro (**a**) depolymerized with DEG and (**b**) depolymerized with DEG+ED (reference polyol is polyol obtained in conventional depolymerization, respectively, with DEG or DEG+ED [24]).

In the case of recycled polyols from foams 400bio and 400petro, a decrease in the previously mentioned peaks characteristic to urethane bonds can also be depicted, but we must take into account bonds characteristic to the isocyanurate ring, specifically -C-N stretching at ~1410 cm^−1^ [30]. In FTIR spectra of recycled polyols from foam 400petro (see Figure 6a,b), a decrease in this peak can be observed, which is in accordance with possible depolymerization. This finding indicates that MW-assisted depolymerization was capable of disrupting not only urethane linkages but also the more thermally stable isocyanurate structures present in PIR foams.

Overall, the FTIR analysis suggests that MW-assisted depolymerization may be effective for both PU and PIR foams. The observed reduction in characteristic urethane and isocyanurate absorption bands is consistent with cleavage of the polymer network. The absence of substantial spectral differences between the investigated processing conditions indicates only limited differences among the tested conditions. The absence of substantial spectral differences between the investigated processing conditions may indicate that comparable degrees of depolymerization could be achieved across the selected MW power levels. These observations are consistent with the hydroxyl value measurements and suggest the potential of MW-assisted depolymerization for the production of recycled polyols.

### 3.4. GPC/SEC Analysis

**Figure 7:**
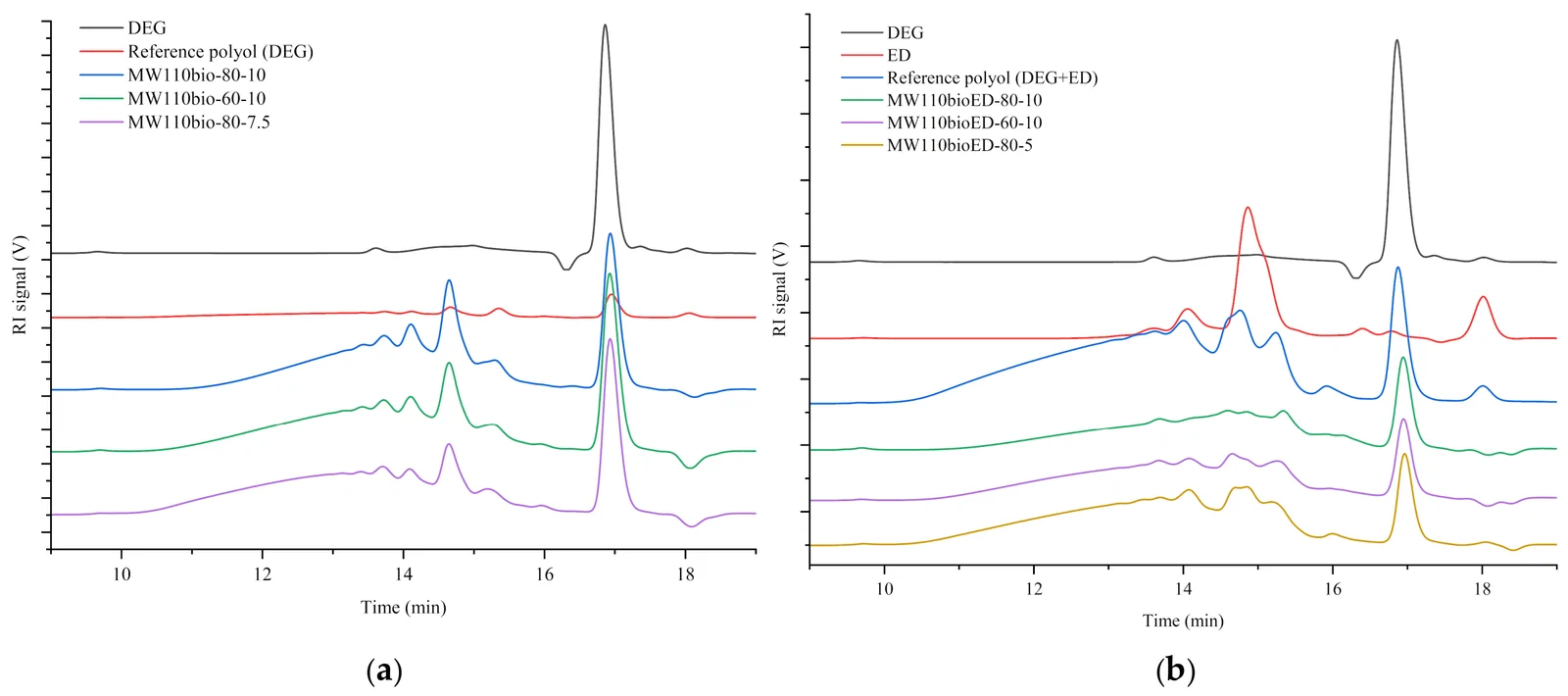
GPC-SEC chromatogram of recycled polyols from foam 110bio (**a**) depolymerized with DEG and (**b**) depolymerized with DEG+ED (reference polyol is polyol obtained in conventional depolymerization, respectively, with DEG or DEG+ED [24]).

In all chromatograms of recycled polyols obtained from foam 110bio (see Figure 7a,b), a pronounced peak at approximately 16.8–17.0 min corresponds to unreacted DEG, indicating that part of the glycolysis agent remained in the recycled polyols after depolymerization. In recycled polyols obtained with DEG+ED (Figure 7b), additional peaks originating from the ED polyol can be observed.

Differences in the GPC/SEC chromatographic profiles were observed between the tested depolymerization conditions. For recycled polyols depolymerized with DEG (Figure 7a), the chromatographic profile shifted towards longer retention times as the reaction proceeded. The first detectable high-molecular-weight fraction appeared at approximately 10.08 min for MW110bio-80-7.5, while it shifted to 10.45 min and 11.05 min for MW110bio-60-10 and MW110bio-80-10, respectively. The shift towards longer retention times is consistent with the presence of lower-molecular-weight species; however, because the GPC/SEC analysis is qualitative and the processing parameters were not independently controlled, the shift cannot be quantitatively related to the extent of depolymerization or to an individual processing parameter.

A similar tendency was observed for recycled polyols depolymerized with DEG+ED (Figure 7b). The first detectable fraction shifted from approximately 10.25 min for MW110bioED-80-5 to 10.55 min and 11.00 min for MW110bioED-60-10 and MW110bioED-80-10, respectively. The broad high-molecular-weight fraction became less pronounced, indicating progressive formation of lower-molecular-weight degradation products. Additional peaks originating from the ED polyol were also observed, resulting in a more complex chromatographic profile than for recycled polyols depolymerized only with DEG.

**Figure 8:**
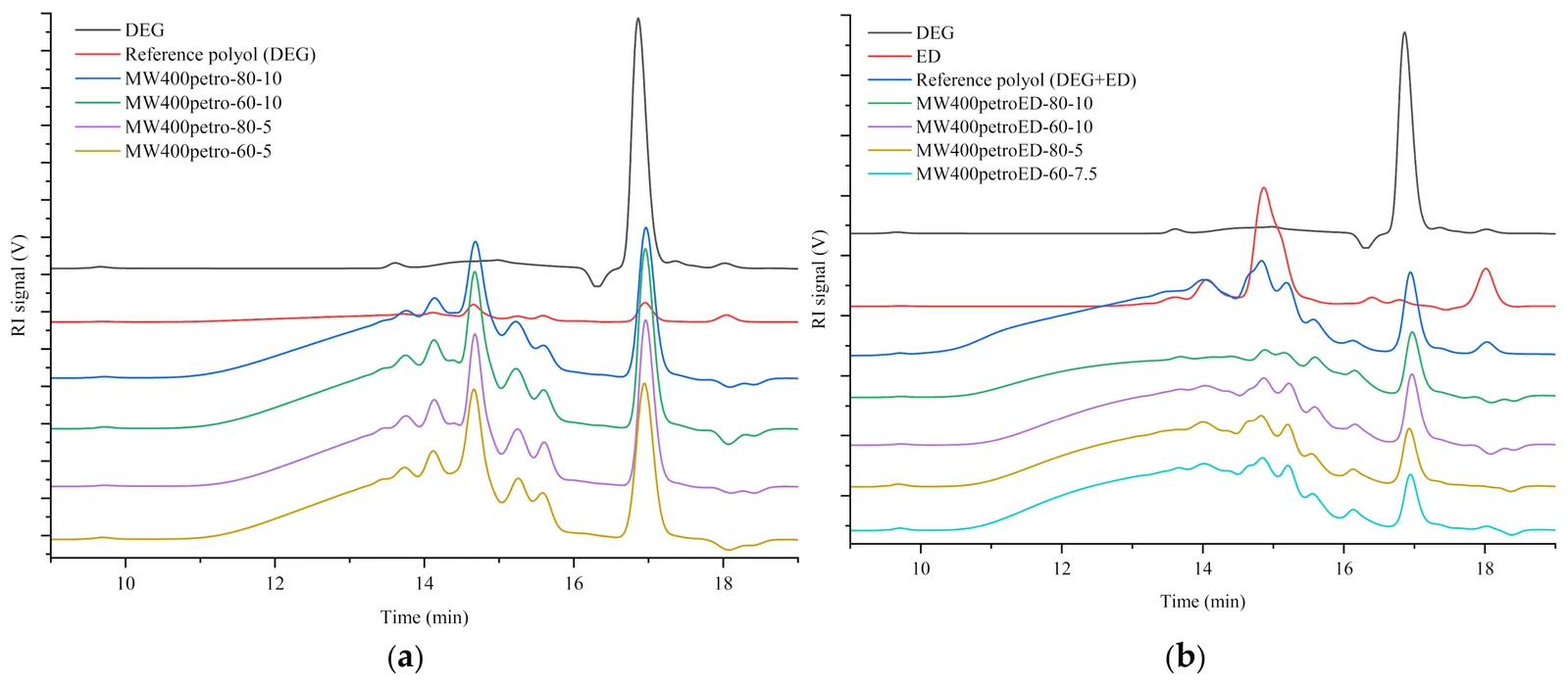
GPC-SEC chromatogram of recycled polyols from foam 400petro (**a**) depolymerized with DEG and (**b**) depolymerized with DEG+ED (reference polyol is polyol obtained in conventional depolymerization, respectively, with DEG or DEG+ED [24]).

Compared to recycled polyols obtained from foam 110bio, chromatograms of recycled polyols from foam 400petro (Figure 8a,b) show only minor differences between the applied depolymerization conditions. The main chromatographic fraction remained within a similar retention-time range across the tested conditions. The less pronounced differences may be associated with the higher isocyanate index of foam 400petro. Similar to 110bio recycled polyols, a distinct DEG peak is observed in all chromatograms, while additional peaks originating from the ED polyol are visible in recycled polyols obtained with DEG+ED.

Conventional GPC/SEC calibration typically uses linear polystyrene standards, which are not fully representative of the complex and branched depolymerization products investigated in this study; therefore, quantitative molecular-weight parameters could not be reliably determined. Therefore, the chromatograms were evaluated qualitatively based on changes in retention time and the relative distribution of different molecular-weight fractions. Chromatograms of other recycled polyols is presented in Supplementary Materials (Figures S5–S8).

### 3.5. Summary of Results

A number of parameters are taken into consideration when characterizing the obtained polyols for additional studies of industrial-scale trials. First, the OH value is one of the most crucial polyol characteristics since it determines how much isocyanate is added to the PU formulation. In addition, our further research will focus on the development of compounds for Michael polymers, and the OH value is one of the key parameters for such experiments. Acid value is particularly important because it can show if the reaction—in this case, depolymerization—has proceeded correctly, and it can affect interaction with isocyanate. Conclusively, viscosity is a crucial factor in polyols because materials with high viscosity are more difficult to exploit at both the laboratory and industrial scale.

In Table 3, we define the range of the mentioned parameters. In Table 4, parameters of the recycled polyols (depolymerized with DEG) are presented.

In Table 4, it can be observed that no polyols show satisfactory results regarding OH values; all results are optimal. This could be adjusted by optimizing reaction conditions. Lower OH values can also be achieved by choosing a different glycolysis agent, which itself has a lower OH value. The majority of the polyols have acceptable AV values; however, there is no discernible correlation between the reaction conditions. Lastly, it must be emphasized that only one polyol shows optimal viscosity, and all others have satisfactory results.

A similar summary of recycled polyols depolymerized with DEG+ED is presented in Table 5.

Opposite to the previous results, here, it can be observed that all recycled polyols have satisfactory OH values despite changing reaction conditions, showing that satisfactory results were obtained under several of the tested conditions. However, AV values of recycled polyols are less desirable; it can be observed that depolymerization of foams 110bio and 110petro for 10 min produces less satisfactory results, which could be due to side reactions occurring during depolymerization. Unfortunately, only few polyols show optimal values of viscosity; others are deficient. This could be due to ED polyols’ high viscosity, and more attractive parameters could be achieved by optimizing reaction conditions.

### 3.6. Depolymerization in Monomodal MW Reactor

Depolymerization of foams was also performed in a monomodal MW reactor, where the reactive mixture was hermetically sealed and the applied power and pressure were precisely monitored. The results from these experiments could help to evaluate the strengths and weaknesses of the depolymerization process for further adaption on an industrial scale. Similar to previously described experiments, depolymerization was performed with DEG and DEG+ED. In Table 6, the conditions of depolymerization are presented.

As depicted in Table 6, foams with an isocyanate index of 400 (400bio and 400petro) depolymerized only with higher foam/polyol mass ratios and with catalyst. Also, the duration of depolymerization during the second part (depolymerizing with DEG+ED polyol) was three-times higher; however, of course, this is still significantly shorter than during conventional depolymerization.

When ED polyol was used for the depolymerization process, the mixture boiled quite fast and pressure rose rapidly (till 10 min); therefore, the duration of depolymerization was extended.

**Figure 9:**
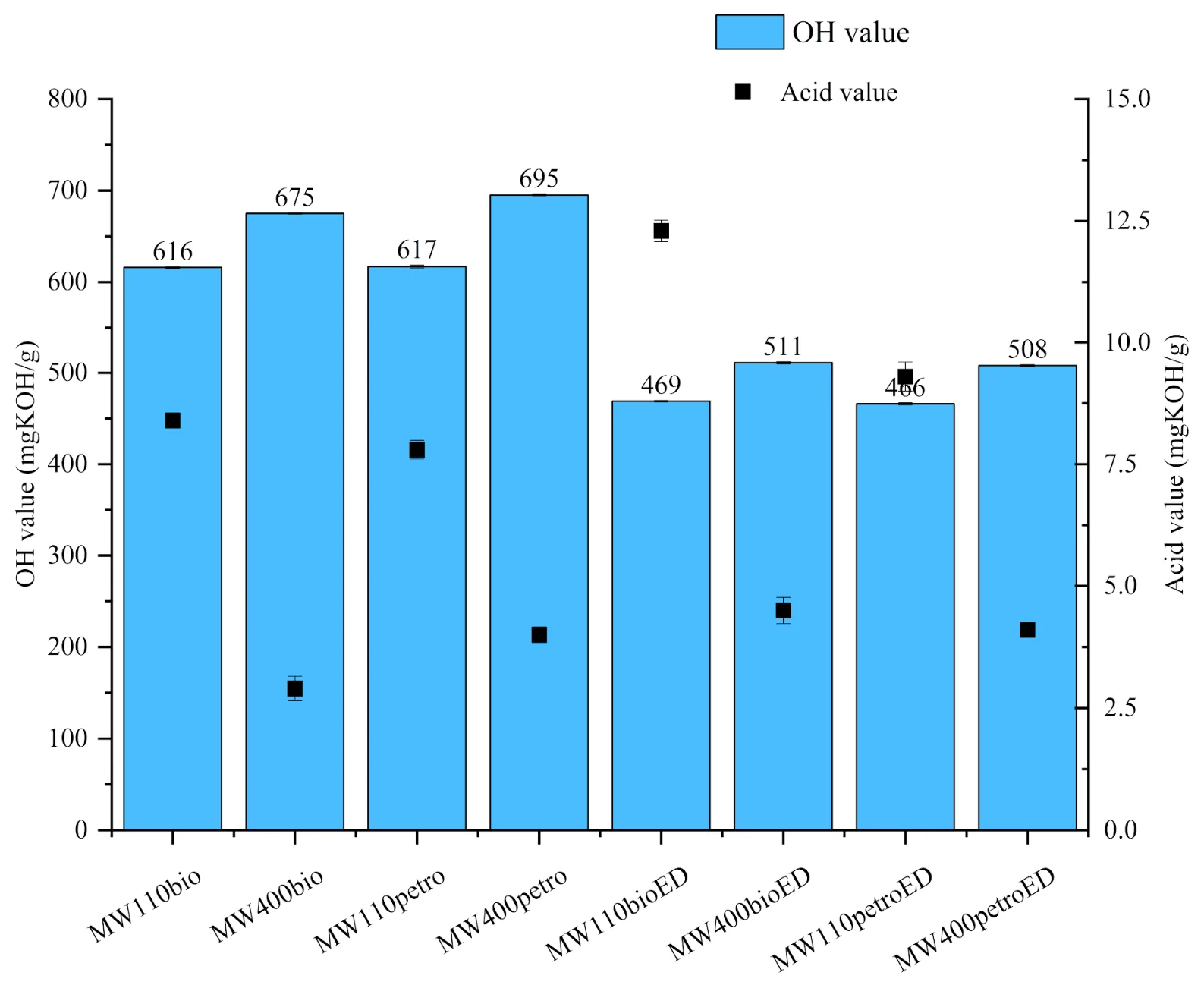
OH values of recycled polyols depolymerized with DEG and DEG+ED in MW reactor.

In Figure 9, OH and acid values of recycled polyols are presented. It can be observed that the OH values of recycled polyols from foams 400bio and 400petro are higher, which is evident because these polyols were obtained with a higher foam/polyol mass ratio (foam/DEG/ED 1:1:1). Nevertheless, these polyols have lower acid values. Lower acid values can be an indication of fewer by-products in the reaction mixture, which can happen because of catalyst KOH. The moisture content of recycled polyols is presented in Supplementary Materials (Table S2).

**Figure 10:**
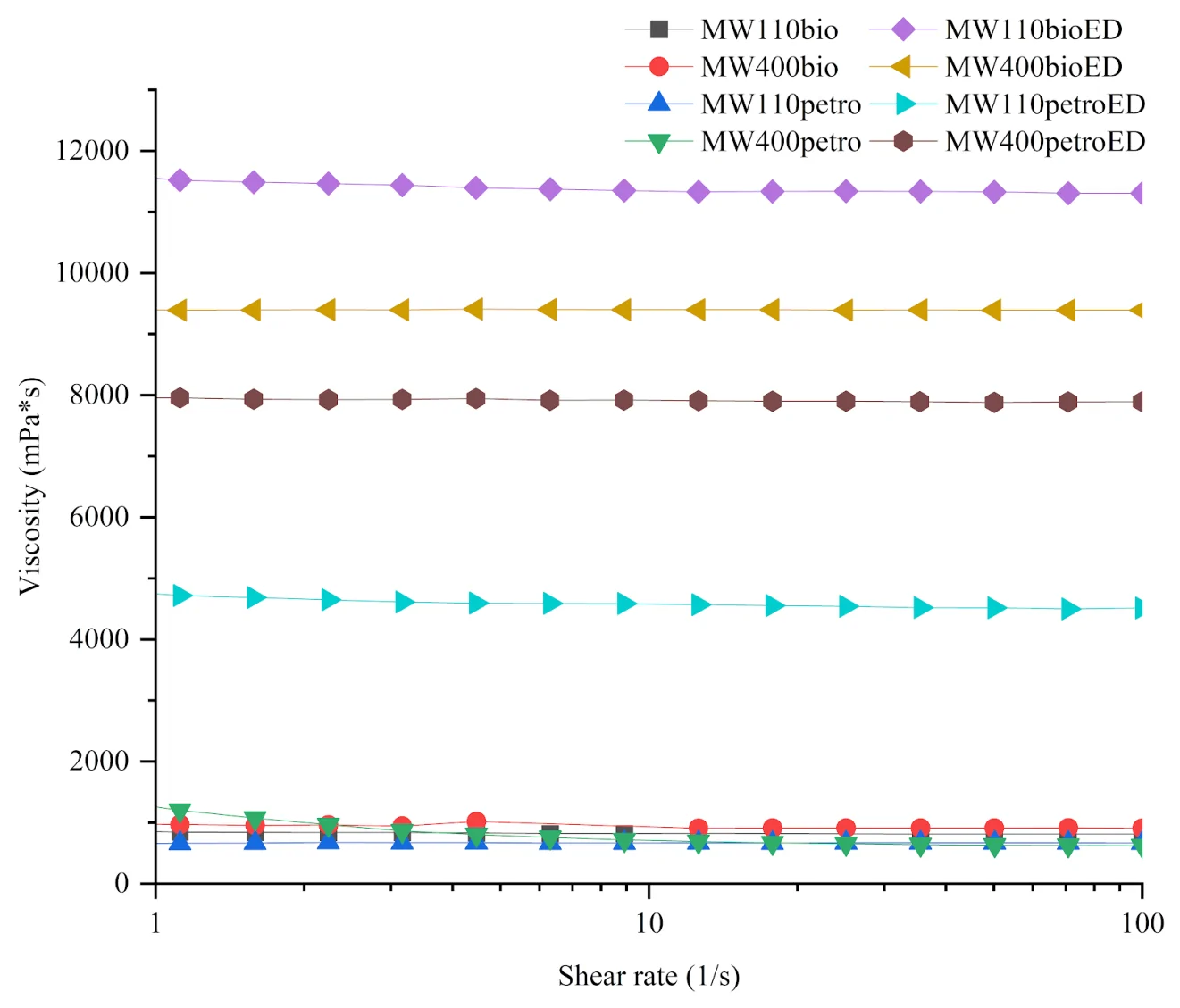
Rheological measurements of recycled polyols depolymerized with DEG and DEG+ED in MW reactor.

As presented in Figure 10, the tested polyols showed approximately Newtonian liquid-type behavior, which, similar to previously obtained recycled polyols, is an appealing characteristic for further laboratory and industrial trials. Recycled polyols which were depolymerized with DEG have lower viscosities because DEG itself has low viscosity. Overall, all recycled polyols have lower viscosities than those which we obtained in the multimodal MW oven. Low viscosities can result from the closed reaction system during depolymerization, which avoids leakage of volatiles.

**Figure 11:**
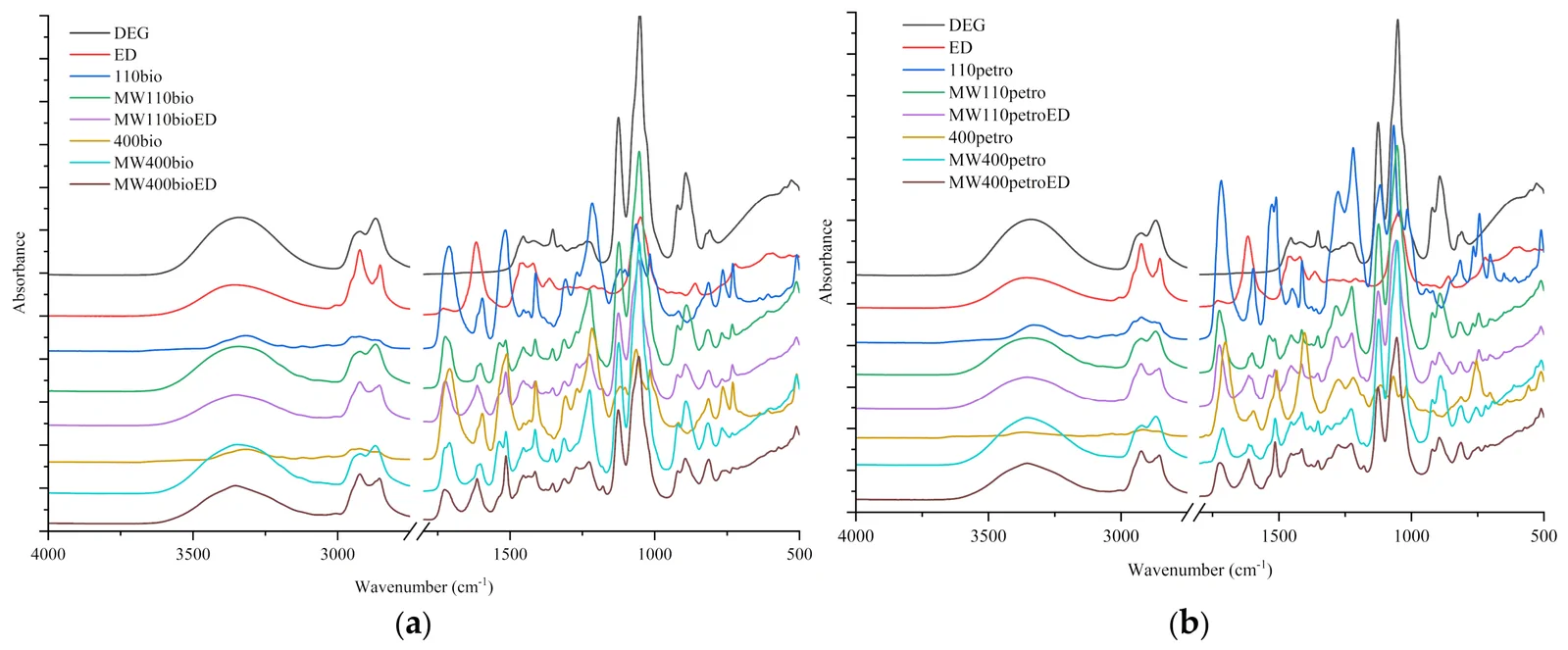
FTIR spectra of recycled polyols from foams (**a**) 110bio and 400 bio and foams (**b**) 110petro and 400petro.

In FTIR spectra of recycled polyols (see in Figure 11), the spectral changes are consistent with chemical changes associated with depolymerization with a decrease in peaks characteristic to urethane groups—carbonyl C=O stretching at ~1720 cm^−1^, -N-H bending at ~1515 cm^−1^ and C-N stretching at ~1600 cm^−1^, as described in previous sections. Also, a decrease in -C-N stretching at ~1410 cm^−1^, which is characteristic to the isocyanurate ring, can be spotted in spectra of recycled polyols from foams 400bio and 400petro. The relative sizes of peaks have also been measured and are presented in Supplementary Materials (Table S3).

**Figure 12:**
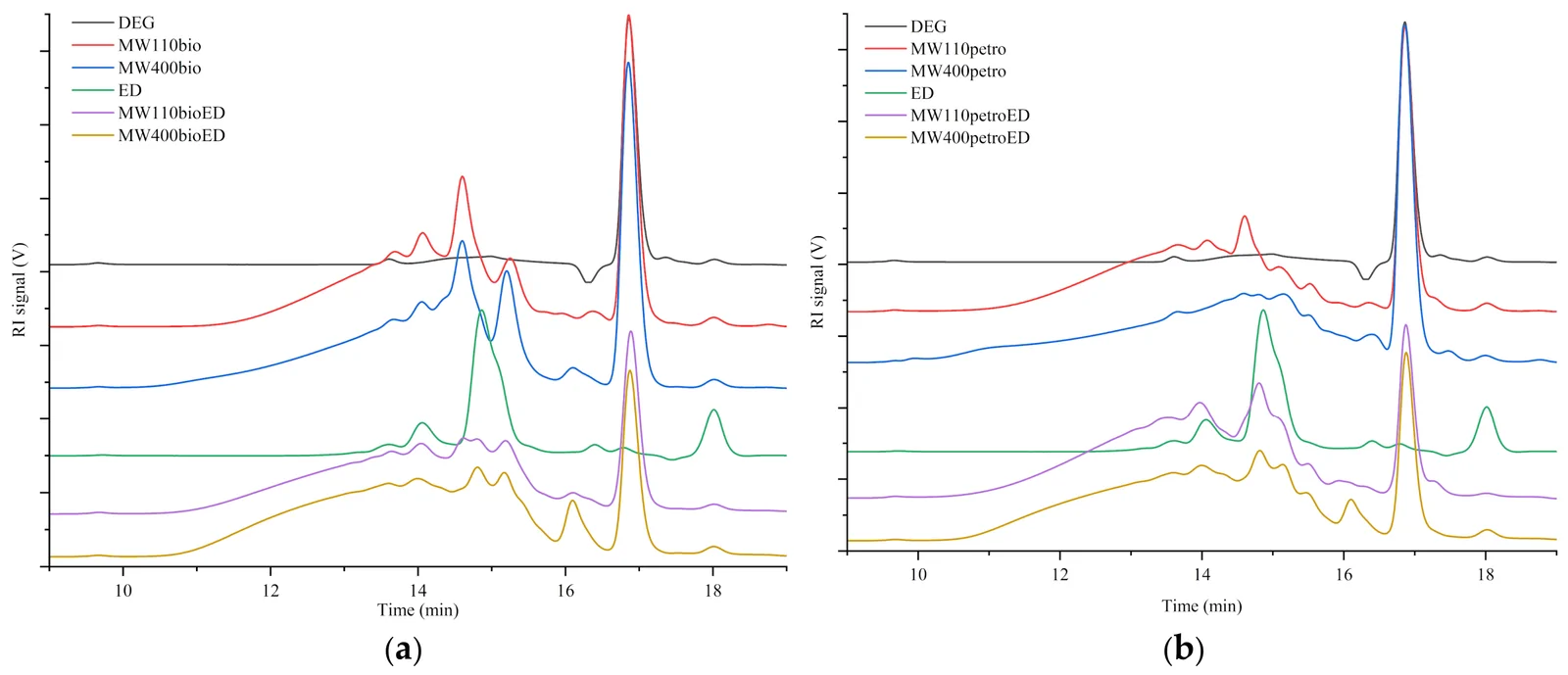
GPC-SEC chromatogram of recycled polyols from foams (**a**) 110bio and 400 bio and foams (**b**) 110petro and 400petro.

GPC/SEC chromatograms (Figure 12a,b) revealed that recycled polyols from foams with an isocyanate index of 400 started to elute at shorter retention times. The chromatographic profile started at approximately 10.20 and 10.45 min for MW400bio and MW400bioED, compared to 11.05 and 10.90 min for MW110bio and MW110bioED, respectively. Similar behavior was observed for petrochemical foams. Furthermore, recycled polyols obtained from foams with an isocyanate index of 400 showed a steeper rise at the beginning of the chromatographic profile. These observations show qualitative differences between the chromatographic profiles of polyols obtained from foams with isocyanate indices of 110 and 400; quantitative differences in molecular-weight distribution cannot be concluded from the present GPC/SEC analysis.

## 4. Conclusions

This study reports the depolymerization of PU and PIR foams through microwave-assisted depolymerization using DEG and epoxidized tall oil-based polyols. Obtained recycled polyols are compared to ones obtained through conventional depolymerization in our previous research.

The first step involved depolymerization with DEG as glycolysis agents through MW irradiation. After analysis of obtained data, it can be concluded that depolymerization occurs at the presented conditions and recycled polyols show promising characteristics; for example, OH values of recycled polyols obtained from foam 110bio were close to reference polyol obtained via conventional depolymerization (587, 608 and 583 mgKOH/g vs. 596 mgKOH/g). Under the tested MW conditions, recycled polyols were obtained within 10 min, compared with 4.5–5.5 h under the previously reported conventional conditions, and in the case of foams with an isocyanate index of 110 (110bio and 110petro), even the catalyst was not necessary.

Substitution of DEG with epoxidized tall oil-based polyol also resulted in recycled polyols with characteristics comparable to the reference polyols obtained through conventional depolymerization, although their viscosity was higher. GPC/SEC analysis qualitatively revealed differences in the chromatographic profiles of the recycled polyols; quantitative molecular-weight parameters were not determined because of the limitations of calibration with linear polystyrene standards for these complex and branched products.

Comparing depolymerization between the MW oven and monomodal reactor, no significant differences can be observed. However, depolymerization in the monomodal MW reactor gives better insight regarding larger-scale MW-assisted depolymerization; for example, the rapid rise of inner pressure is one of the factors that must be considered.

The results demonstrate that MW-assisted depolymerization can produce recycled polyols under the tested conditions within substantially shorter reaction times than the conventional process used for comparison; however, energy consumption was not evaluated in this study. The obtained recycled polyols can be successfully used for the development of Michael monomers and used for further polymer formulations.

## Figures and Tables

**Table 1:** MW-assisted depolymerization conditions depolymerizing with DEG.

Foam	Recycled Polyol	Catalyst	Foam/DEG Mass Ratio	Power Level, %	Time, min	Temperature, °C
110bio	MW110bio-80-10	-	1:1.5	80	10	~180
	MW110bio-60-10			60	10	~180
	MW110bio-80-7.5			80	7.5	~140
400bio	MW400bio-80-10	KOH		80	10	~170
	MW400bio-60-10			60	10	~150–160
	MW400bio-80-5			80	5	~140
	MW400bio-60-7.5			60	7.5	~140
110petro	MW110petro-80-10	-		80	10	~170
	MW110petro-60-10			60	10	~150
	MW110petro-80-5			80	5	~130
	MW110petro-60-7.5			60	7.5	~130
400petro	MW400petro-80-10	KOH		80	10	~170
	MW400petro-60-10			60	10	~150–160
	MW400petro-80-5			80	5	~160
	MW400petro-60-5			60	5	~130

**Table 2:** MW-assisted depolymerization conditions depolymerizing with DEG and ED polyol.

Foam	Recycled Polyol	Catalyst	Foam/DEG/ED Mass Ratio	Power Level, %	Time, min	Temperature, °C
110bio	MW110bioED-80-10	-	1:0.75:0.75	80	10	~160
	MW110bioED-60-10			60	10	~160
	MW110bioED-80-5			80	5	~140
400bio	MW400bioED-80-10	KOH		80	10	~160
	MW400bioED-60-10			60	10	~190
	MW400bioED-80-5			80	5	~120–130
	MW400bioED-60-7.5			60	7.5	~120–130
110petro	MW110petroED-80-10	-		80	10	~150
	MW110petroED-60-10			60	10	~155
	MW110petroED-80-5			80	5	~140
	MW110petroED-60-5			60	5	~140
400petro	MW400petroED-80-10	KOH		80	10	~160
	MW400petroED-60-10			60	10	~155–160
	MW400petroED-80-5			80	5	~130
	MW400petroED-60-7.5			60	7.5	~150

**Table 3:** Range of OH value, acid value and viscosity.

	Deficient ●	Optimal ●	Satisfactory ●
OH value OH, mgKOH/g	>700	500–700	<500
Acid value AV, mgKOH/g	>5	4–5	<4
Viscosity µ, mPa·s	>40,000	10,000–40,000	<10,000

**Table 4:** Parameters of the recycled polyols in MW-assisted depolymerization (depolymerized with DEG).

	110											400												
bio	Ratio	1:1.5										Ratio	1:1.5											
	Time, min	10					7.5					Time, min	10			7.5				5				
	Char.	OH		AV	µ		OH		AV	µ		Char.	OH	AV	µ	OH		AV	µ	OH		AV		µ
	80%	●		●	●		●		●	●		80%	●	●	●	-		-	-	●		●		●
	60%	●		●	●		-		-	-		60%	●	●	●	●		●	●	-		-		-
petro	Ratio	1:1.5										Ratio	1:1.5											
	Time, min	10			7.5				5			Time, min	10						5					
	Char.	OH	AV	µ	OH	AV		µ	OH	AV	µ	Char.	OH		AV		µ		OH		AV		µ	
	80%	●	●	●	-	-		-	●	●	●	80%	●		●		●		●		●		●	
	60%	●	●	●	●	●		●	-	-	-	60%	●		●		●		●		●		●	

**Table 5:** Parameters of the recycled polyols in MW-assisted depolymerization (depolymerized with DEG+ED).

	110							400									
bio	Ratio	1:0.75:0.75						Ratio	1:0.75:0.75								
	Time, min	10			7.5			Time, min	10			7.5			5		
	Char.	OH	AV	µ	OH	AV	µ	Char.	OH	AV	µ	OH	AV	µ	OH	AV	µ
	80%	●	●	●	●	●	●	80%	●	●	●	-	-	-	●	●	●
	60%	●	●	●	-	-	-	60%	●	●	●	●	●	●	-	-	-
petro	Ratio	1:0.75:0.75						Ratio	1:0.75:0.75								
	Time, min	10			7.5			Time, min	10			7.5			5		
	Char.	OH	AV	µ	OH	AV	µ	Char.	OH	AV	µ	OH	AV	µ	OH	AV	µ
	80%	●	●	●	●	●	●	80%	●	●	●	-	-	-	●	●	●
	60%	●	●	●	●	●	●	60%	●	●	●	●	●	●	-	-	-

**Table 6:** Conditions of depolymerization process in MW reactor.

Foam	Recycled Polyol	Catalyst	Foam/DEG Mass Ratio	Time, min	Power, W	Temperature, °C
110bio	MW110bio	-	1:1.5	10	150	140–160
400bio	MW400bio	KOH	1:1	5		
110petro	MW110petro	-	1:1.5	10		
400petro	MW400petro	KOH	1:1	10		
			**Foam:DEG:ED mass ratio**			
110bio	MW110bioED	-	1:0.75:0.75	10	150	140–160
400bio	MW400bioED	KOH	1:1:1	30		
110petro	MW110petroED	-	1:0.75:0.75	6		
400petro	MW400petroED	KOH	1:1:1	30		

## Data Availability

The authors state that the presented data will be available on request by email.

## Supplementary Materials

The following supporting information can be downloaded at https://www.mdpi.com/article/10.3390/polym18202498/s1, Table S1. Moisture content and acid value of recycled polyols; Table S2. Moisture content of recycled polyols (depolymerised in MW reactor); Table S3. Relative sizes of peaks characteristic to polyurethane bond of recycled polyols; Figure S1. FTIR spectra of recycled polyols from foam 110petro depolymerised with DEG; Figure S2. FTIR spectra of recycled polyols from foam 110petro depolymerised with DEG+ED; Figure S3: FTIR spectra of recycled polyols from 400bio depolymerized with DEG; Figure S4. FTIR spectra of recycled polyols from foam 400bio depolymerised with DEG+ED; Figure S5. GPC/SEC spectra of recycled polyols from foam 110petro depolymerised with DEG; Figure S6. GPC/SEC spectra of recycled polyols from foam 110petro depolymerised with DEG+ED; Figure S7. GPC/SEC spectra of recycled polyols from foam 400bio depolymerised with DEG; Figure S8. GPC/SEC spectra of recycled polyols from foam 400bio depolymerised with DEG+ED.

## Abbreviations

The following abbreviations are used in this manuscript:

DEG	diethylene glycol
DEOA	diethanolamine
ETOFA	epoxidized tall oil fatty acids
ETOFA/DEOA or ED	epoxidized tall oil fatty acids/diethanolamine polyol
MW	microwave
PIR	polyisocyanurate
PU	polyurethane
TOFA	tall oil fatty acids
