# Supplementary material for: Microwave-Assisted Depolymerization of Polyurethane and Polyisocyanurate Foams with Diethylene Glycol and Epoxidized Tall Oil-Based Polyol
Source: Polymers (Basel). 2026 Oct 13;18(20):2498. doi: 10.3390/polym18202498 (PMC13709248; doi:10.3390/polym18202498)

Supplementary material contents: Table S1 – acid value and moisture content of recycled polyols obtained in the multimodal MW oven; Table S2 – moisture content of recycled polyols obtained in the monomodal MW reactor; Table S3 – relative sizes of FTIR peaks characteristic of polyurethane and isocyanurate bonds; Figures S1–S4 – FTIR spectra of the additional recycled polyol samples; Figures S5–S8 – GPC/SEC chromatograms of the additional recycled polyol samples. Each dataset and figure is identified individually below.

Table S1. Moisture content and acid value of recycled polyols.

Foam	Recycled polyol	Acid value, mgKOH/g	Moisture content, %
110bio	MW110bio-80-10	5.4	0.19
	MW110bio-60-10	4.4	0.15
	MW110bio-80-7.5	3.0	0.18
	MW110bioED-80-10	5.4	0.4
	MW110bioED-60-10	6.1	0.18
	MW110bioED80-5	3.8	0.37
400bio	MW400bio-80-10	2.2	0.11
	MW400bio-60-10	2.3	0.11
	MW400bio-80-5	0.8	0.13
	MW400bio-60-7.5	1.3	0.17
	MW400bioED-80-10	3.4	0.73
	MW400bioED-60-10	2.8	0.54
	MW400bioED80-5	0.6	0.37
	MW400bioED60-7.5	1.0	0.32
110petro	MW110petro-80-10	5.8	0.12
	MW110petro-60-10	4.8	0.14
	MW110petro-80-5	1.1	0.21
	MW110petro-60-7.5	1.5	0.13
	MW110petroED-80-10	6.3	0.2
	MW110petroED-60-10	4.8	0.29
	MW110petroED80-5	2.8	0.60
	MW110petroED60-5	2.1	0.47
400petro	MW400petro-80-10	3.7	0.01
	MW400petro-60-10	4.7	0.11
	MW400petro-80-5	0.9	0.15
	MW400petro-60-5	1.2	0.11
	MW400petroED-80-10	3.3	0.65
	MW400petroED-60-10	3.8	0.48
	MW400petroED80-5	1.0	0.68
	MW400petroED60-7.5	1.3	0.56

Table S2. Moisture content of recycled polyols (depolymerised in MW reactor).

Foam	Recycled polyol	Moisture content
110bio	MW110bio	0.78

	MW110bioED	0.95
400bio	MW400bio	0.27
	MW400bioED	0.71
110petro	MW110petro	0.47
	MW110petroED	0.88
400petro	MW400petro	0.89
	MW400petroED	1.26

Table S3. Relative sizes of peaks characteristic to polyurethane bond of recycled polyols

	**Bond:** C=O (~1705), 1760-1640 cm^{-1}		**Bond:** N-H (~1515), 1560-1470 cm^{-1}		**Bond:** C-N (~1600), 1630-1570 cm^{-1}		**Bond:** isocyanurate C-N (~1410), 1430-1390 cm^{-1}	
**Sample**	**Size**	**%**	**Size**	**%**	**Size**	**%**	**Size**	**%**
**DEG**	0.66		2.03		0.47		3.12	
**110bio**	16.85	100.00	15.72	100.00	4.98	100.00	4.94	100.00
MW110bio	7.13	42.31	7.34	46.69	2.59	52.01	3.81	77.13
MW110bioED	5.94	35.25	6.38	40.59	3.65	73.29	3.54	71.66
MW110bio-80-10	9.08	53.89	9	57.25	3.03	60.84	4.16	84.21
MW110bio-60-10	8.54	50.68	8.45	53.75	2.83	56.83	4.07	82.39
MW110bio-80-7.5	8.90	52.82	8.95	56.93	3.03	60.84	4.15	84.01
MW110bioED-80-10	5.71	33.89	6.86	43.64	4.33	86.95	3.54	71.66
MW110bioED-60-10	8.03	47.66	8.22	52.29	4.7	94.38	3.95	79.96
MW110bioED-80-5	8.1	48.07	8.09	51.46	4.35	87.35	4.06	82.19
**400bio**	15.98	100.00	14.64	100.00	4.95	100.00	5.62	100.00
MW400bio	6.87	42.99	8.05	54.99	2.78	56.16	4.49	79.89
MW400bioED	5.76	36.05	7.02	47.95	3.94	79.60	4.15	73.84
MW400bio-80-10	10.07	63.02	12.00	81.97	4.02	81.21	4.76	84.70
MW400bio-60-10	10.13	63.39	12.14	82.92	4.03	81.41	4.56	81.14
MW400bio-80-5	9.88	61.83	11.73	80.12	3.89	78.59	4.61	82.03
MW400bio-60-7.5	9.51	59.51	11.11	75.89	3.71	74.95	4.58	81.49
MW400bioED-80-10	6.4	40.05	9.24	63.11	5.15	104.04	4.08	72.60
MW400bioED-60-10	7.33	45.87	7.75	52.94	3.98	80.40	3.78	67.26
MW400bioED80-5	10.11	63.27	10.50	71.72	4.98	100.61	4.65	82.74
MW400bioED60-7.5	10.7	66.96	11.86	81.01	5.70	115.15	5.08	90.39

	**Bond:** C=O (~1705), 1760-1640 cm^{-1}		**Bond:** N-H (~1515), 1560-1470 cm^{-1}		**Bond:** C-N (~1600), 1630-1570 cm^{-1}		**Bond:** isocyanurate C-N (~1410), 1430-1390 cm^{-1}	
**Sample**	**Size**	**%**	**Size**	**%**	**Size**	**%**	**Size**	**%**
**110petro**	22.28	100.00	19.26	100.00	6.37	100.00	4.97	100.00
MW110petro	7.49	33.62	5.89	30.58	1.99	31.24	3.46	69.62
MW110petroED	7.98	35.82	5.97	31.00	3.49	54.79	3.6	72.43
MW110petro-80-10	8.34	37.43	6.54	33.96	2.23	35.01	3.56	71.63
MW110petro-60-10	8.16	36.62	6.46	33.54	2.19	34.38	3.56	71.63
MW110petro-80-5	7.58	34.02	6.03	31.31	2.03	31.87	3.53	71.03
MW110petro-60-7.5	7.42	33.30	5.85	30.37	1.98	31.08	3.42	68.81
MW110petroED-80-10	6.7	30.07	4.89	25.39	3.38	53.06	2.96	59.56
MW110petroED-60-10	8	35.91	5.87	30.48	3.62	56.83	3.35	67.40
MW110petroED80-5	8.53	38.29	6.38	33.13	3.97	62.32	3.66	73.64
MW110petroED60-5	8.27	37.12	6.25	32.45	3.99	62.64	3.70	74.45
**400petro**	14.86	100.00			3.71	100.00	8.69	100.00
MW400petro	4.85	32.64			2.02	54.45	4.29	49.37
MW400petroED	6.14	41.32			3.7	99.73	4.18	48.10
MW400petro-80-10	10.33	69.52			3.64	98.11	4.56	52.47
MW400petro-60-10	9.94	66.89			3.51	94.61	4.42	50.86
MW400petro-80-5	7.51	50.54			2.92	78.71	3.75	43.15
MW400petro-60-5	9.78	65.81			3.54	95.42	4.27	49.14
MW400petroED-80-10	6.96	46.84			4.38	118.06	3.78	43.50
MW400petroED-60-10	9.38	63.12			4.69	126.42	4.20	48.33
MW400petroED80-5	10.46	70.39			5.22	140.70	4.52	52.01
MW400petroED60-7.5	10.83	72.88			5.41	145.82	4.56	52.47

Figure S1. FTIR spectra of recycled polyols from foam 110petro depolymerised with DEG.

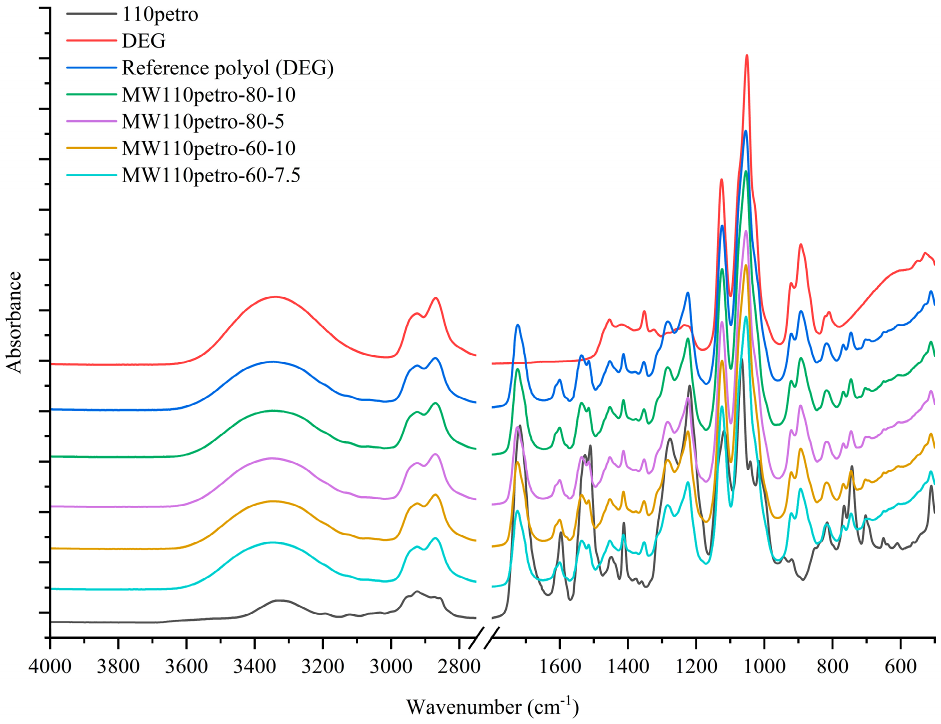

Figure S2. FTIR spectra of recycled polyols from foam 110petro depolymerised with DEG+ED.

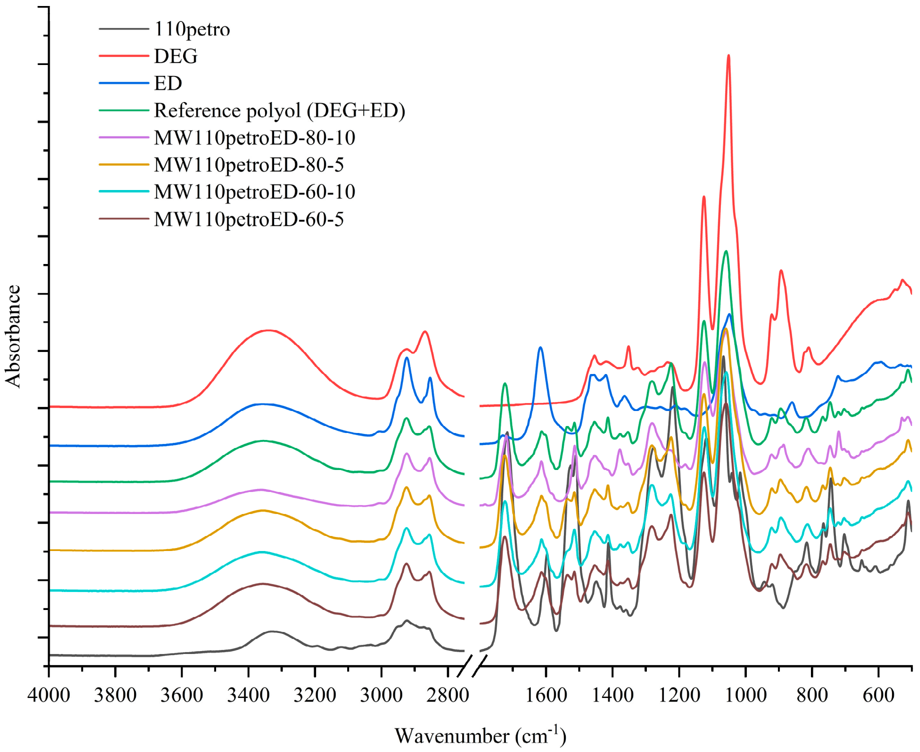

Figure S3. FTIR spectra of recycled polyols from foam 400bio depolymerised with DEG.

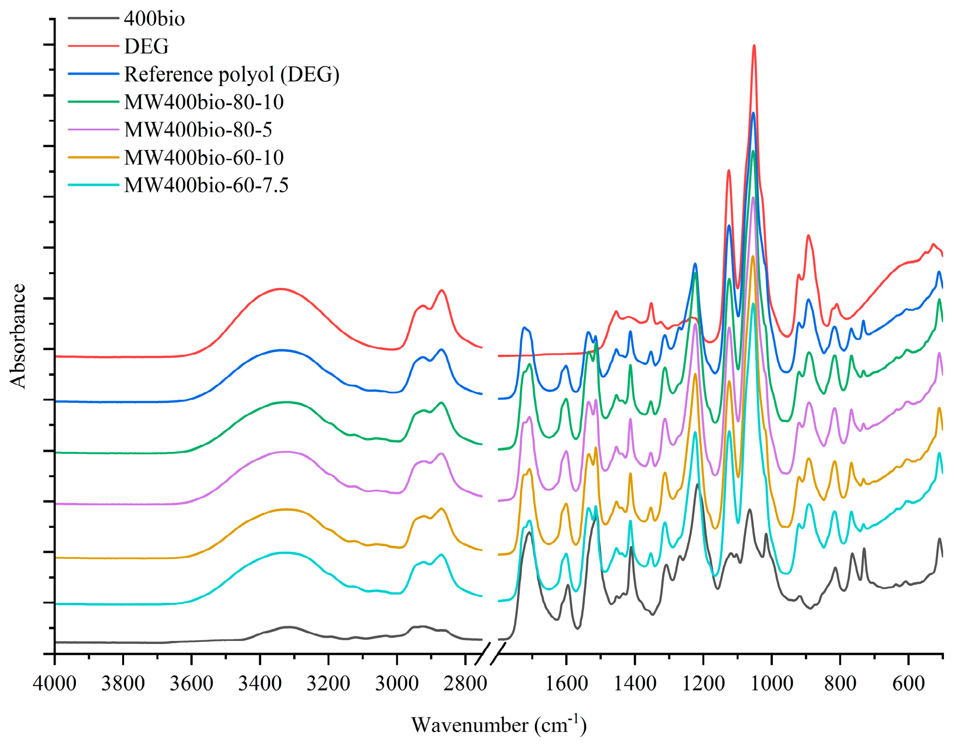

Figure S4. FTIR spectra of recycled polyols from foam 400bio depolymerised with DEG+ED.

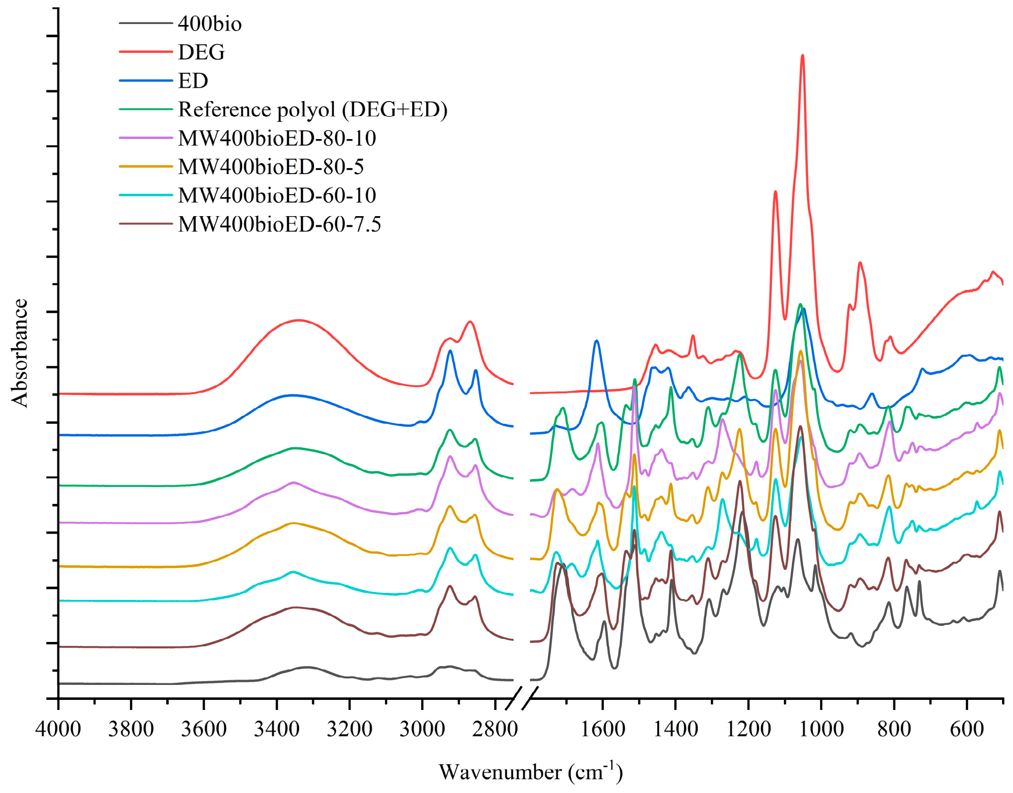

Figure S5. GPC/SEC spectra of recycled polyols from foam 110petro depolymerised with DEG.

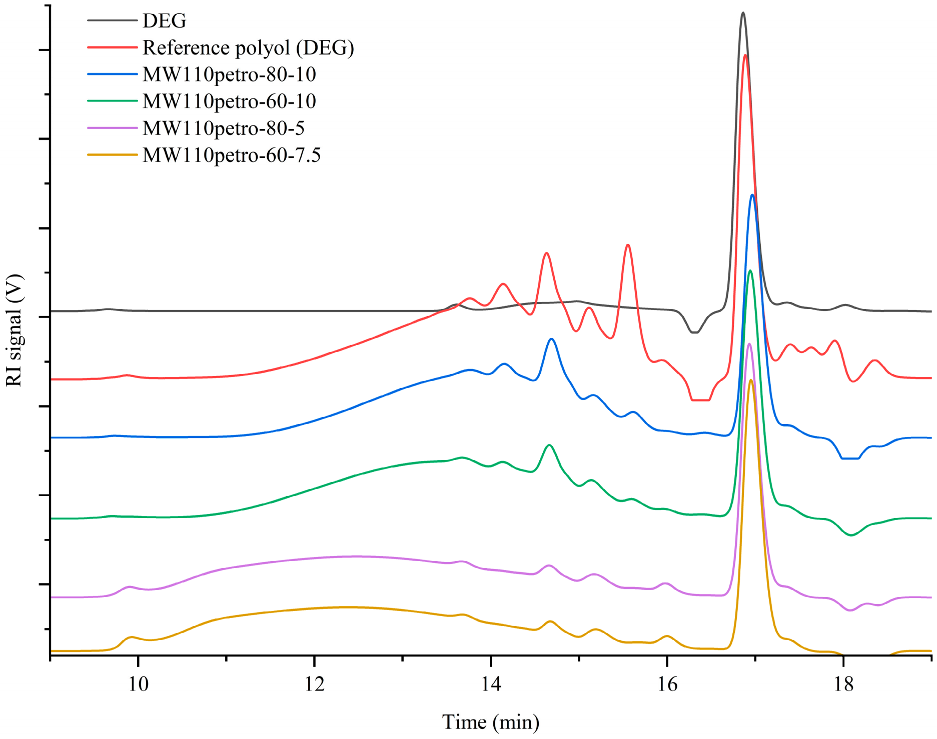

Figure S6. GPC/SEC spectra of recycled polyols from foam 110petro depolymerised with DEG+ED.

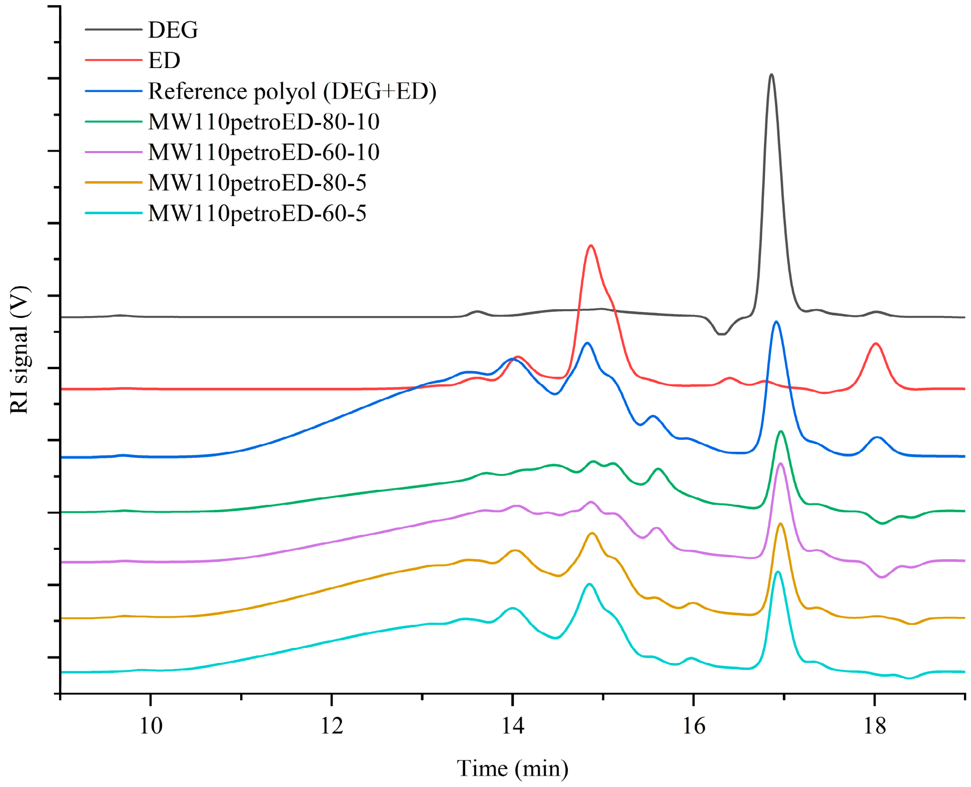

Figure S7. GPC/SEC spectra of recycled polyols from foam 400bio depolymerised with DEG.

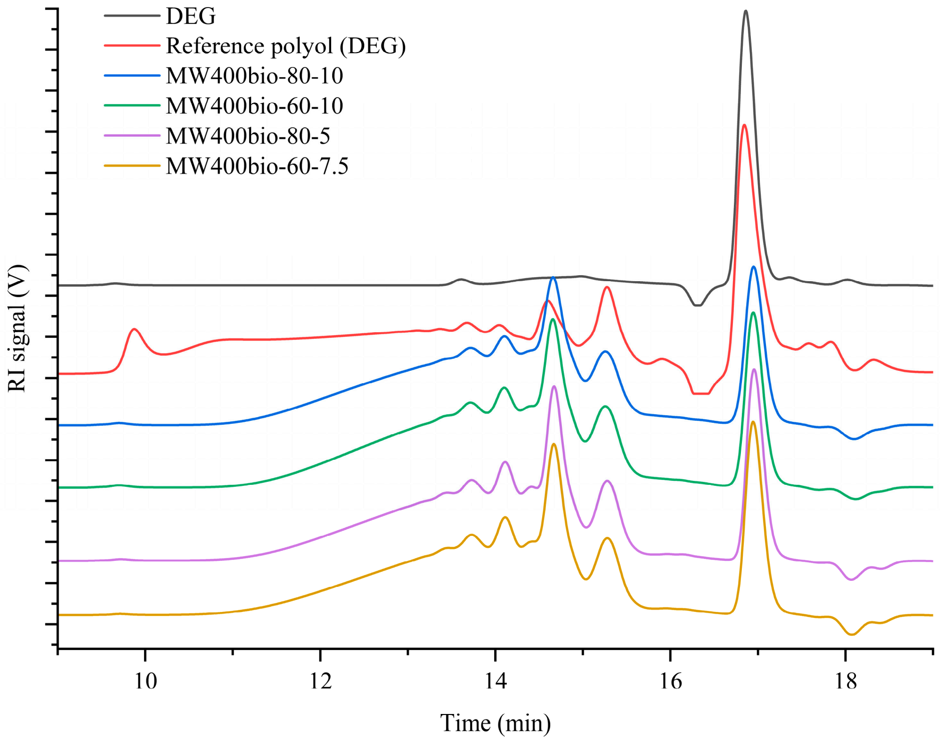

Figure S8. GPC/SEC spectra of recycled polyols from foam 400bio depolymerised with DEG+ED.

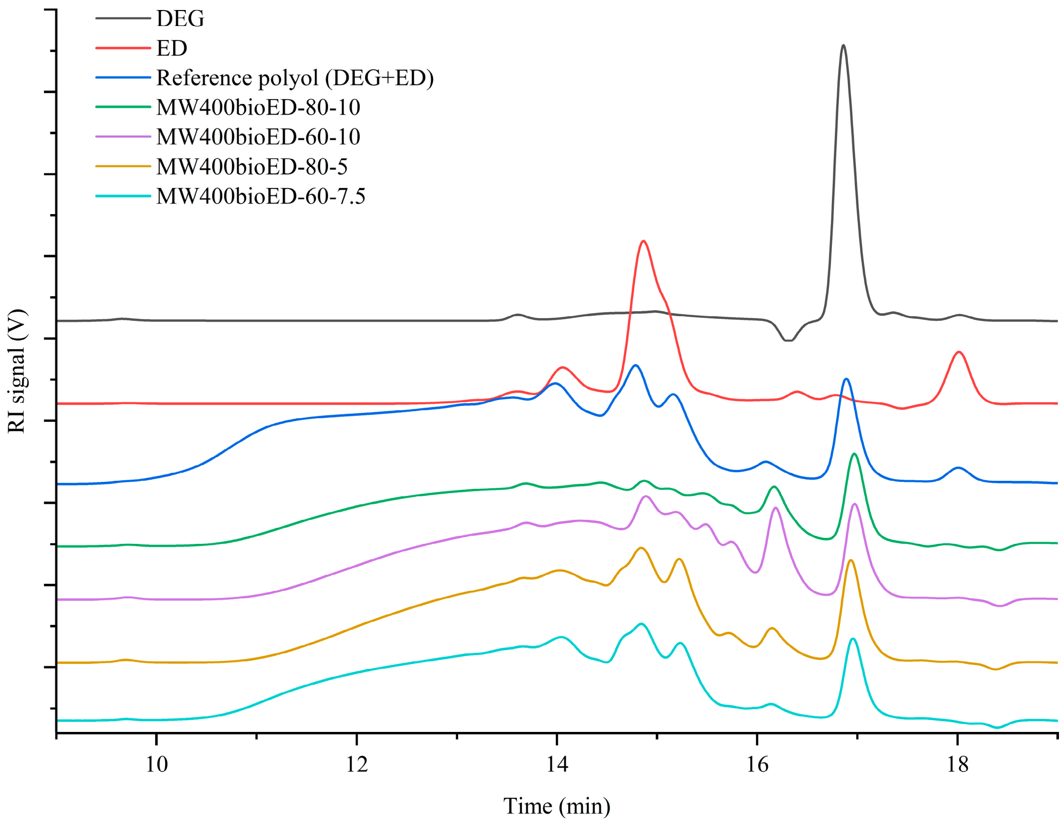

Supplement: Supplementary file 1 [file polymers-18-02498-s001.zip › polymers-4519117-supplementary.pdf]
